# Sequestration Effect on the Open-Cyclic Switchable Property of Warfarin Induced by Cyclodextrin: Time-Resolved Fluorescence Study

**DOI:** 10.3390/molecules22081326

**Published:** 2017-08-11

**Authors:** Naji Al-Dubaili, Na’il Saleh

**Affiliations:** Chemistry Department, College of Science, United Arab Emirates University, Al-Ain 15551, UAE; n.dubaili@uaeu.ac.ae

**Keywords:** open-cyclic tautomers, molecular switching, decay-associated spectra, warfarin, excited-state lifetime, cyclodextrins

## Abstract

The excited-state lifetimes of the anticoagulant drug warfarin (W) in water and in the absence and presence of methyl-β-cyclodextrins (Me-β-CD) were recorded using time-resolved fluorescence measurements. Selective excitation of the open and cyclic protonated isomers of W were acquired with laser emitting diodes (LED) producing 320 and 280 nm excitation pulses, respectively. Formation of the inclusion complex was checked by UV-visible absorption spectroscopy, and the values of binding constants (2.9 × 10^3^ M^–1^ and 4.2 × 10^2^ M^−1^ for protonated and deprotonated forms, respectively) were extracted from the spectrophotometric data. Both absorption and time-resolved fluorescence results established that the interior of the macromolecular host binds preferentially the open protonated form, red shifts the maximum of its absorption of light at ~305 nm, extends its excited-state lifetime, and decreases its emission quantum yield (Ф_F_). Collectively, sequestration of the open guest molecules decreases markedly their radiative rate constants (*k*_r_), likely due to formation of hydrogen-bonded complexes in both the ground and excited states. Due to lack of interactions, no change was observed in the excited-state lifetime of the cyclic form in the presence of Me-β-CD. The host also increases the excited-state lifetime and Ф_F_ of the drug deprotonated form (W^−^). These later findings could be attributed to the increased rigidity inside the cavity of Me-β-CD. The p*K*_a_ values extracted from the variations of the UV-visible absorption spectra of W versus the pH of aqueous solution showed that the open isomer is more acidic in both ground and excited states. The positive shifts in p*K*_a_ values induced by three derivatives of cyclodextrins: HE-β-CD, Ac-β-CD, and Me-β-CD supported the preferential binding of these hosts to open isomers over cyclic.

## 1. Introduction

**Scheme 1:**
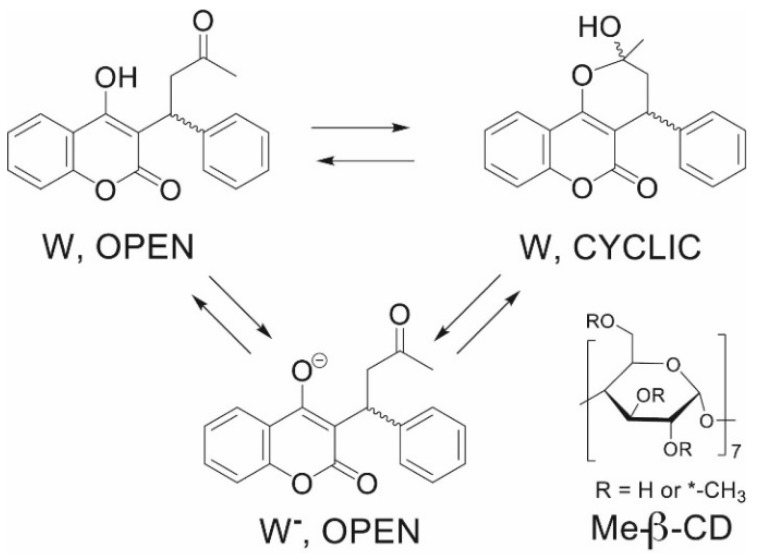
Structural formulas of protonated warfarin (W): open and cyclic isomers, deprotonated warfarin (W^−^), and methyl-β-cyclodextrins (Me-β-CD) molecular container.

The fluorescence properties of coumarin derivatives have attracted the attention of organic physical chemists for several decades [1]. Our research group, in particular, synthesized two coumarin derivatives for fluorescent sensing of pH [2]. Like other fluorescent probes, coumarins display photophysical properties that are sensitive to their local environments, such as the presence of supramolecular host cavities [3]. Within this context, we have also exploited a supramolecular host-guest approach alongside fluorescence behavior of a third coumain derivative, demonstrating a new sensing method for molecular recognition of optically inactive analytes [4]. During the course of our research, we realized the need to characterize the photophysical behaviors of warfarin (W) inside the cavities of cyclodextrin (CD) macromolecular hosts (Scheme 1). W, which is one of the known fluorescent coumarin derivatives, is also a highly potent anticoagulant drug commonly prescribed as COUMADIN^®^ for the control and prevention of blood clots [5].

There have been number of studies on the structures of W in different solvents and at different pH values by NMR, UV-visible absorption and time-resolved fluorescence [6,7,8,9]. Early studies have concluded that the structure of drug in solution at pHs lower than its p*K*_a_ ~5 presents as either open or cyclic protonated form (predominantly as the structures in Scheme 1) and at pH higher than its p*K*_a_, as a deprotonated form (W^−^; Scheme 1), whose side chain is open [6,7]. In addition to that, the photophysical properties of W in different solvents and solvent mixtures were profoundly investigated [8,9]. Results confirmed that the protonated open form absorbs at *λ* = 310 nm, whereas the cyclic form peak appears at *λ* = 280 nm. The results were then exploited to examine the binding of W to human serum albumin (HSA) [8], as well as model systems such as CDs [9]. In the later study, only steady-state fluorescence measurements have been undertaken, which warrants a more quantitative tool for examining the effects of supramolecular cavities. Accordingly, measurements of fluorescence lifetimes of W in the absence and presence of CDs at different pH are highly motivated. 

In this article, time-resolved fluorescence data of W have been collected as a function of pH and excitation wavelengths in water and inside β-CD molecular containers. We observed that excited-state lifetime of W increases upon binding to methyl-β-cyclodextrin (methyl-β-CD) (Scheme 1) and we were able to selectively monitor the interaction of each protonated tautomer (open vs. cyclic) with the host molecules. We have specifically demonstrated while the length of excited-state lifetimes of the CD-encaged W^−^ depends on the effective viscosity of the surrounding microenvironment, extended fluorescence lifetime of the open protonated form of W upon enclathration is explained by the radiative-rate law. The difference in acidity between the two isomers of W is also experimentally investigated in the ground and excited state. 

## 2. Results and Discussion

### 2.1. Interactions of Warfarin with Cyclodextrins

**Figure 1:**
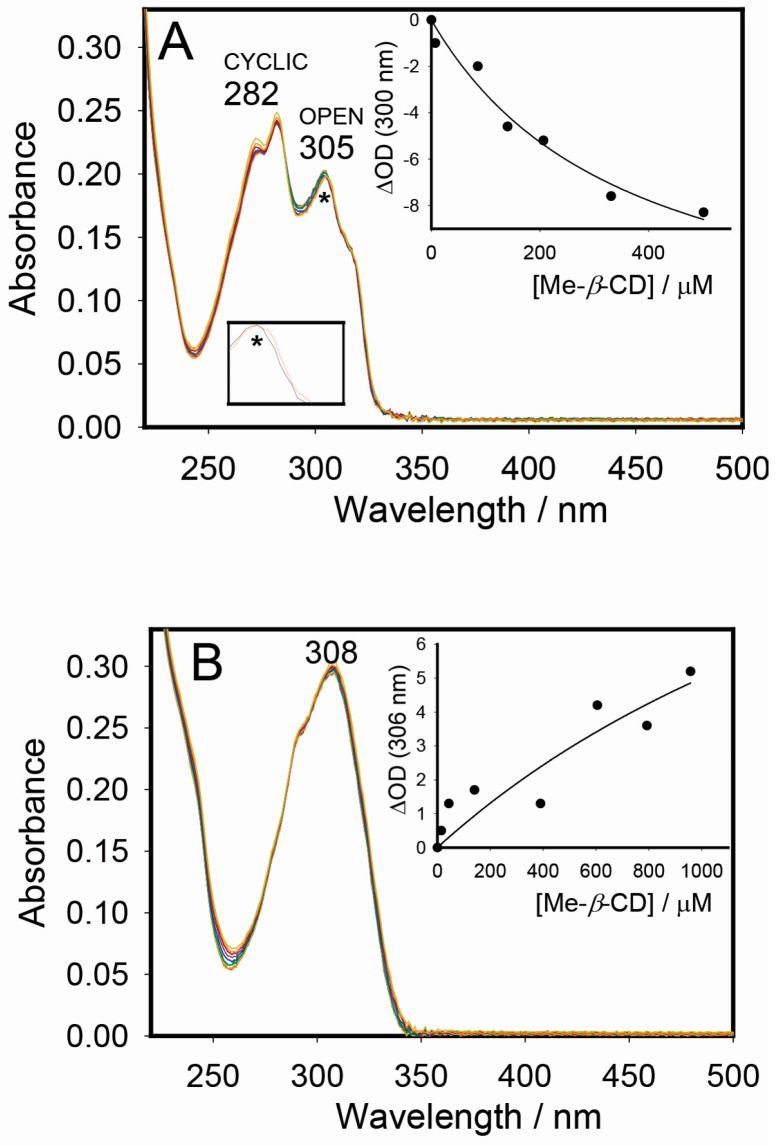
UV-visible absorption titration of W (25 μM) with Me-β-CD at pH 3 (**A**), and pH 9 (**B**); the *inset* shows the corresponding titration curve and the 1:1 binding fit (solid line) with *K* = (2.9 ± 0.3) × 10^3^ M^‒1^, and *K* = (4.2 ± 1.0) × 10^2^ M^‒1^, respectively.

Preceding studies on the interactions of W with several CD derivatives have been taken into consideration while planning the experiments for this part [9,10,11,12]. Accordingly, new derivatives of β-CD were selected, namely Ac-β-CD and HE-β-CD (Chart S1, Supporting Information). The rationale behind our selection arises from the presence of additional hydroxyl or carbonyl functional groups that could enhance interactions with W. Unfortunately, both macrocycles gave weak or no interactions with W, as monitored by UV-visible absorption measurements at pH 3 and 9 (Figure S1, Supporting Information). However, the previously examined derivative Me-β-CD gave the highest binding constants [10], as illustrated in Figure 1. The lack of encapsulation by other β-CD cages is due to size mismatch, more flexible cages and weaker non-bonded interactions with hydroxycoumarin derivatives [3]. The data in Figure 1 were not published before and it was necessary to present them here to preclude the lifetime measurements below. Two pH values (3 and 9) were selected at which W persists as the protonated and deprotonated form (W^−^), respectively (see pH-titration results below). In Figure 1A at pH 3, the acyclic open protonated form binds moderately to the host (*K* = 2900 M^–1^), whereas W^−^ binds weakly to CD at high pH (Figure 1B) with a binding constant of 420 M^–1^ in agreement with previous reports [9,11,12]. Low binding affinities of coumarins to CD systems are not surprising because the presence of hydroxyl group is known to hinder inclusion. Upon addition of Me-β-CD host molecules (up to 40 equivalents) to the aqueous solutions of W at pH 3 and 9 (Figure 1), characteristic changes in the UV-visible absorption spectra were observed with the occurrence of several isosbestic points (311 nm, 315 nm, and 320 nm) confirming the formation of a 1:1 binding stoichiometry. The corresponding binding constants between W and Me-β-CD at a given pH were derived directly from the optical titration plot at a given wavelength using the formula described in the Experimental section.

Earlier report in octanol/water model system at pH 7.4 concluded that cytochrome P450 2C9 (CYP2C9) stabilized the cyclic form of W [13]. Absorption spectra upon addition of different amounts of β-CD to W in water at pH 7.4 (phosphate buffer) were later measured by Vasquez et al. [9]. Those authors concluded that W exists predominantly in its open form when bound to β-CD. They attributed this observation to a steric factor that forces W to remain in its open structure. Our findings at pH 3 support selective interactions of CD with the open form, during which only the longest absorption wavelength at 305 nm of the protonated W embedded in β-CD was affected (red shift from 305 to 306 nm), probably for similar steric reasons to those suggested by Vasquez et al. [9].

### 2.2. Optical Measurements and Host-Induced pK_a_ Shifts

**Figure 2:**
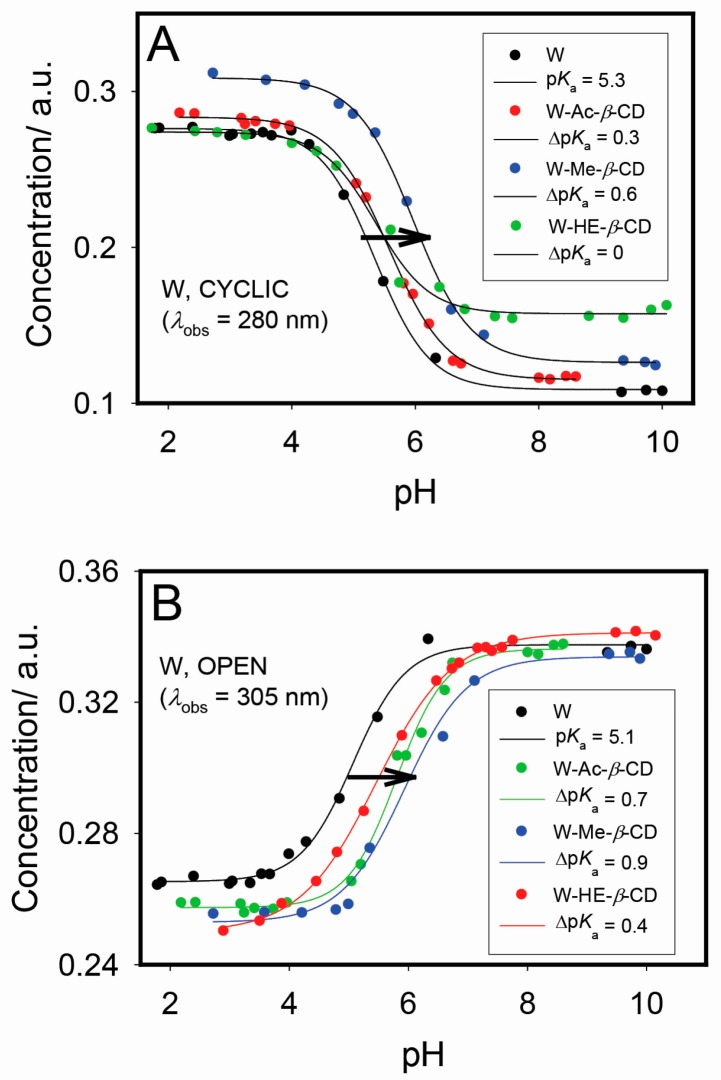
UV-visible absorption spectra of W in aqueous solutions at different pH values from 2–10 in water and inside the cavities of HE-β-CD, Ac-β-CD, and Me-β-CD. The experimental fitting errors for the labeled p*K*_a_ values and the corresponding spectra are shown in Figure S2, Supporting Information.

A very recent experimental and theoretical investigations by Nowak et al. on several hydroxycouamrin derivatives concluded that different hydroxyl group locations should affect the value of p*K*_a_ [14]. Motivated by this work, we looked at the changes of UV-visible absorption spectral profiles of free and CD-bound W in aqueous solutions as a function of pH values (Figure S2, Supporting Information). Me-β-CD-induced p*K*_a_ shifts corresponding to the deprotonation processes of the hydroxyl group in the coumarin ring (Scheme 1) have already been studied before by Nowak et al. yet our inspiration here specifically comes from the expected dependence of the extracted p*K*_a_ values on the selected wavelength in the corresponding titration plots (see Experimental section) [10]. Accordingly, repeating these experiments besides those corresponding to the new β-CD derivatives should enforce our understanding whether changing the position of hydroxyl group in the open and cyclic isomers would affect their p*K*_a_ values or not. Figure 2 illustrates the extracted pH titration plots for the cyclic (280 nm) and open (305 nm) W forms from data in Figure S2, Supporting Information. Positive shifts were observed upon addition of all CD derivatives in aqueous solution under similar conditions of ionic strength effects (see experimental section) [15]. Regardless of the type of isomer, Me-β-CD-assisted p*K*_a_ shifts are always larger than those induced by Ac-β-CD (Δp*K*_a_ ~0.9 vs. ~0.7 for open forms and ~0.6 vs. ~0.3 for cyclic forms).The HE-β-CD induced the least p*K*_a_ shifts (Δp*K*_a_ ~0.4 for open forms, and no shift observed in case of cyclic). 

The host-induced p*K*_a_ shifts reflect the changes in the binding affinities of drug to β-CD derivatives in the ground states (see Me-β-CD as an example at pH 3 and 9 in Figure 1), rationalized by preferential interactions of host towards the protonated form over the deprotonated one [16]. Similarly, the difference in the low binding constant values of the other two β-CD cages to each W species (although indicating no significant inclusion) is reflected in the increase in p*K*_a_ (Figure 2) and thus attributed to the preference for binding of host to protonated W species over deprotonated ones [4,16].

Nowak et al. [10] attributed the strongest p*K*_a_ shifts in Me-β-CD to the presence of a methyl group that may have preferentially interacted with the CD cavity. More important to the focus of our paper is the observation that sequestration of drug into the three β-CD hosts increased the p*K*_a_ of the open isomer more than that of cyclic form, supporting the selective complexation with open form as concluded above. In addition to that, the open form of free W appears to be more acidic in ground state (p*K*_a_ 5.1 vs. 5.3), presumably due to the extended electron delocalization in the final charged product (W^−^) [14].

### 2.3. Excitation, pH, and Cyclodextrin Dependence of Warfarin Steady-State Fluorescence

**Figure 3:**
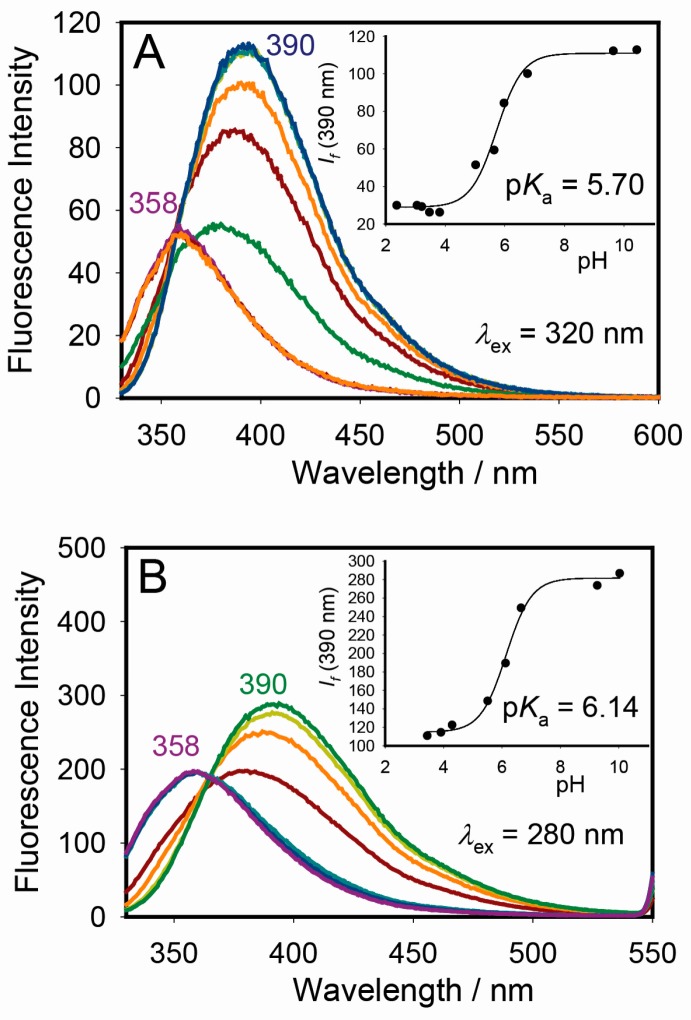
Fluorescence spectra of W (25 μM) at different pH values with excitation at 320 nm (**A**); and 280 nm (**B**); the *inset* shows the experimental fit to a sigmoidal function (solid line), which gives p*K*_a_ = 5.70 ± 0.07, and 6.14 ± 0.05, respectively. Slit widths were 5 nm for excitation and 10 nm for emission monochromators in spectra B.

Our paper specifically aimed at investigating the dependence of fluorescence of W on pH and excitation wavelength in the absence and presence of Me-β-CD, the host which has sufficiently interacted with W by virtue of the above absorption measurements. Fluorescence pH titration experiments were performed as illustrated in Figure 3. Different p*K*_a_ values were obtained upon exciting W in water at 280 and 320 nm (p*K*_a_ 6.1 vs. 5.7), which is attributed to the deprotonation of the hydroxyl proton (Scheme 1), with the open form being more acidic than the cyclic one.

This could be ascribed to extended electron delocalization in the excited-state structure of the corresponding deprotonated form in parallel to the behaviors of isomers in the ground-state [14]. It must be noted that open and cyclic forms, despite having similar emission maxima, gave different emission profiles when excited at 280 nm instead of 320 nm (Figure S3, Supporting Information), confirming the persistence of intramolecular proton transfer from the open form to the cyclic tautomer (Scheme 1) in the excited state. 

**Figure 4:**
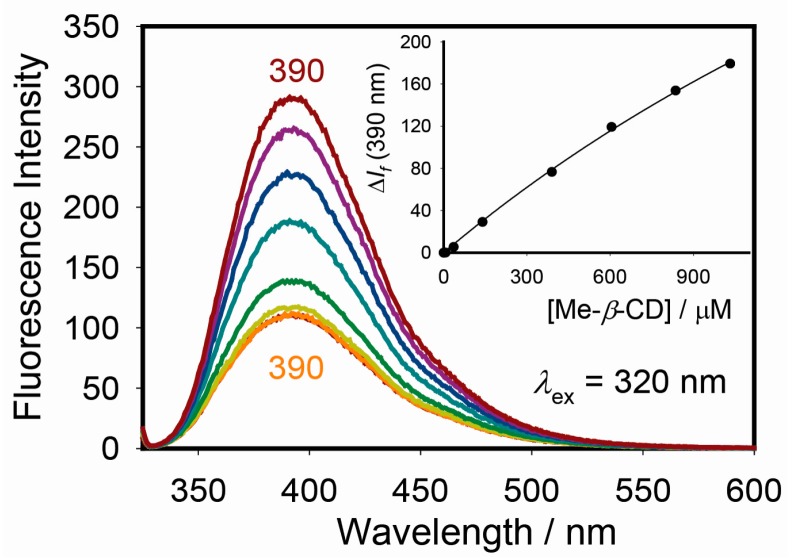
Fluorescence titration of W^−^ (25 μM) with Me-β-CD at pH 9 with excitation at 320 nm the inset shows the corresponding titration curve and the 1:1 binding fit (solid line) with *K* = (2.66 ± 0.06) × 10^2^ M^−1^.

As far as the effects of CDs on emission of W, we observed a three-fold fluorescence enhancement of the encaged W^−^ by Me-β-CD at pH 9 (Figure 4; excitation at 320 nm) with binding affinity of 266 M^−1^ in agreement previous reports [9,11,12]. However, the protonated (open or cyclic) forms at pH 3 showed very weak enhancement in fluorescence upon addition of 10 equivalents of host (Figure S4, Supporting Information). Such contradictory results warranted further investigation using time-resolved fluorescence spectroscopy. 

### 2.4. Fluorescence Lifetime Measurements/Decay-Associated Spectra (DAS)

We resorted to time-resolved fluorescence measurements to rationalize the lack of fluorescence enhancement of protonated W upon incorporation in Me-β-CD. We also sought to separate the complexation effects of host molecules on each protonated isomer (open vs. cyclic) by monitoring the change in excited-state lifetimes of W upon selective excitation of each form at 320 and 280 nm. In previous reports in organic solvents, the two forms were simultaneously excited at 300 nm [8]. Emission decays collected every 10 nm across the entire emission spectra of free W at pH 3 upon excitation at 320 and 280 were globally fitted to a monoexponential decay function, as shown in Figures S5 and S6 in the Supporting Information and tabulated in Table 1.

**Figure 5:**
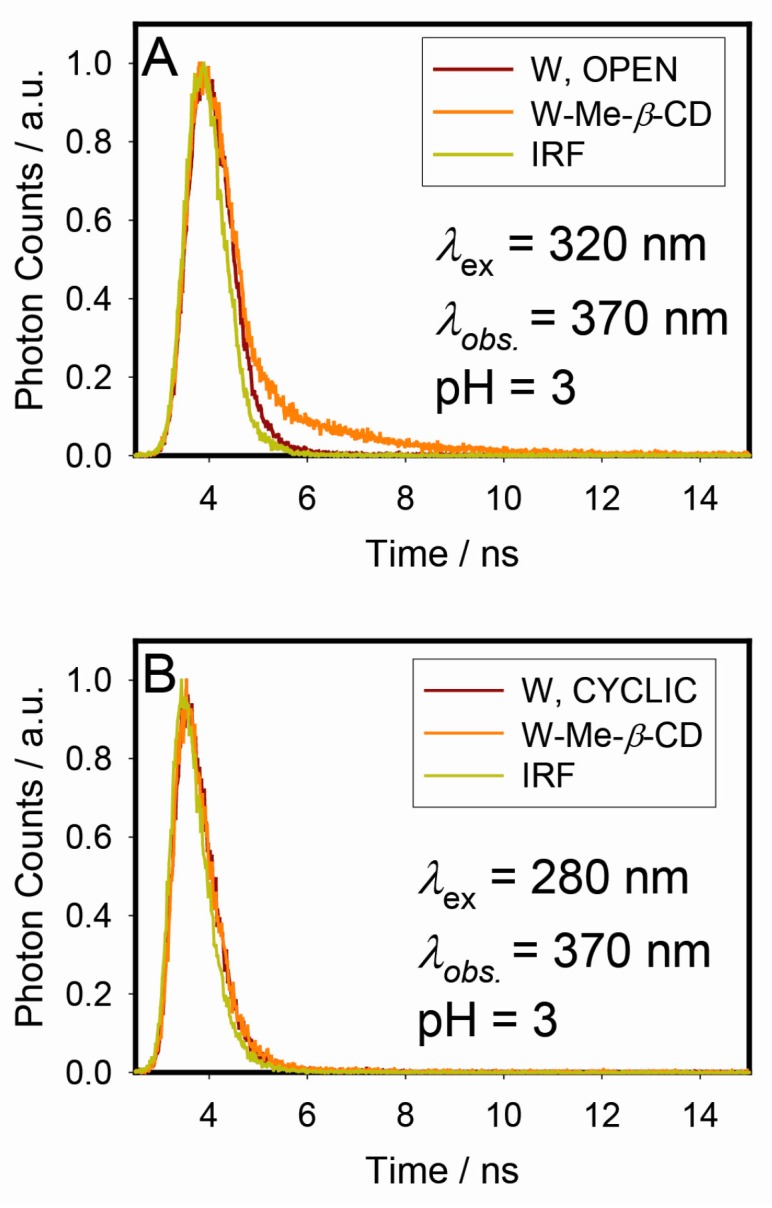
Emission decays collected at 370 nm for W at 25 µM in the absence and presence of Me-β-CD (1.0 mM) upon exciting at 280 nm (**A**) and 320 nm (**B**). No change in fluorescence lifetime upon excitation at 280 nm. IRF is the instrument response function of ~90 ps.

The excited-state lifetimes of free W appear within our instrument resolution of ~90 ps in agreement with similar reports [8,17]. However, when Me-β-CD complexes were excited at pH 3 both monoexponential and biexponential decays were observed (Figures S7 and S8, Supporting Information). Although addition of CD at pH 3 did not affect the position of peaks (390 nm), the excited-state lifetime increased, but only when the complex was excited at 320 nm, not 280 (Figure 5, Figures S7 and S8, Supporting Information), supporting that Me-β-CD host favors the open tautomer form. Even though we collected emission decays of drugs in Figure 5 upon addition of 40 equivalents of host (limited by its solubility in water of about 10 mM), complete formation of complex has not been achieved due to its relatively weak binding constants. 

**Figure 6:**
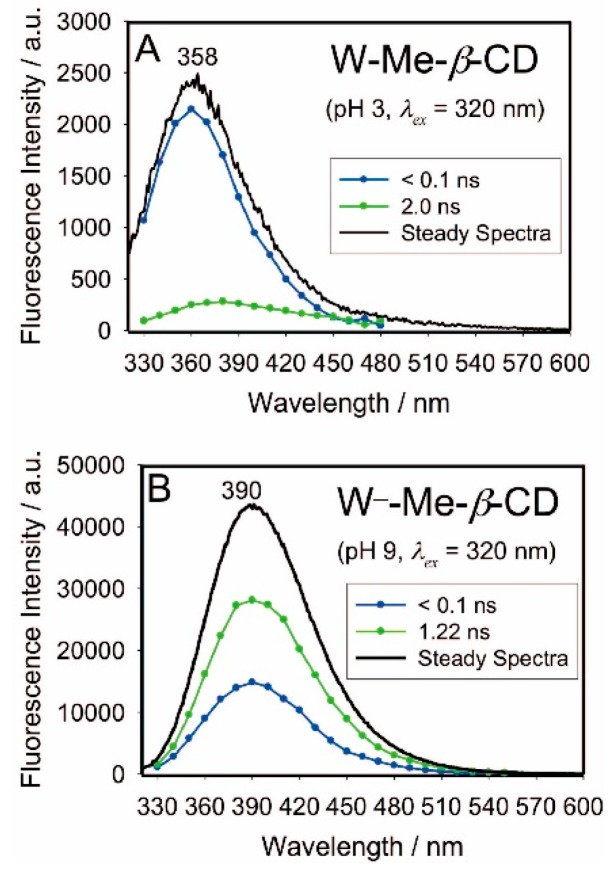
Decay-associated spectra (DAS) of two-component mixture of fluorophores for W-Me-β-CD host-guest complex (25 μM for W, and 1 mM for W-Me-β-CD) at pH 3 (**A**) and pH 9 (**B**) and upon excitation at 320 nm and room temperature. The corresponding steady-state spectra of each solution are also shown for comparison (see experimental section).

Accordingly, the corresponding DAS spectra in Figure 6 (see Experimental section) are best interpreted by assuming contributions from both free and CD-complexed drug. The emission of free drug at pH 3 dominates the emission spectrum with an emission band centered at 358 nm (Figure 6A). The corresponding complex at this pH has an emission peak at 375 nm with a longer excited-state lifetime of 2.0 ns, yet much lower emission quantum yields (0.006 vs. 0.0005 in Table 2). 

Association of each extracted lifetime to each species by the DAS method enables us to track the changes in the corresponding radiative (*k*_r_) and non-radiative rate (*k*_nr_) constants alongside emission quantum yields (Ф) upon complexation of W to Me-β-CD, as illustrated in Table 2. The variations of *k*_r_ values in the absence and presence of CD are difficult to explain because of the unreliable measurement of lifetimes in water. However, the red shifts of emission peaks of W upon complexation to Me-β-CD hosts at pH 3 despite their non-polar hydrophobic and rigid cavity along with concomitant significant decrease of the *k*_r_ values by ~2 orders of magnitude, from *k*_r_ = ~6 × 10^7^ to 2 × 10^5^ s^‒1^, means that the enhancement in excited-state lifetimes is best described by a radiative-rate law [18].

This argument is supported by the boarder UV-visible spectrum of W when compared to that spectrum in water upon inclusion to CD, as shown in Figure 1A and in light of Strickler-Berg equation [18]. Karlsson et al. [8] pointed out the possibility of attributing the longer lifetime of W observed in ethanol (0.45 ns, *λ*_ex_ = 295 nm) to the formation of solute-solvent hydrogen-bonded complexes. Dondon et al. [17] also posited that for other 4-hydroxycoumarin derivatives similar hydrogen-bonded complexes could have formed between the coumarin lactone group and the CD secondary hydroxyl groups. It transpires that there is a plausible explanation to the fluorescence behaviors of protonated W-CD systems that similar hydrogen-bonded complexes have formed between CD secondary hydroxyl group and the carbonyl group of open form, which the cyclic form lacks. It is worth to mention that the low binding affinity of our W-CD system in the present study limits its potential use for biomedical applications as a large fraction of free guest will always persist at any concentration of the added host. However, understanding the factors that enhance the binding constants of W with CDs such as the formation of hydrogen bonded complexes [8,17] and the presence of methyl groups [10] alongside the investigation of pH effects on the complex stability should preclude further studies in the future on W-CD systems for the establishment of pH-driven stimuli-responsive systems.

Emission decays collected at pH 9 (Figures S9 and S10, Supporting Information) gave DAS spectra (Figure 6B) that demonstrated opposite effects induced by the addition of macromolecular host. In agreement with previous reports [9,11,12], the complex at pH 9 excited at 320 nm has a higher emission yield than that of free drug with excited-state lifetime of 1.22 ns and no change in peak position at 390 nm (Table 2). Vasquez et al. suggested encapsulation of deprotonated W^−^ forms by Me-β-CD suppresses considerably the vibronic modes that provide pathways for non-radiative transitions between the excited and ground states of W, causing a decrease in the rates of the non-radiative decay processes. Indeed, our calculation supports these findings within the context of energy-gap law. The *k*_nr_ values of W in Table 2 decreased ~12 times upon inclusion in the hydrophobic interior of Me-β-CD, whereas *k*_r_ values decrease only ~6 times. The increase in lifetimes and associated decrease in *k*_nr_ of other 4-hydroxycoumarin derivatives [17] from below 0.5 ns to 1.1–1.3 ns (depending on the derivative) were also observed upon sequestration of dyes into β-CD macromolecules.

## 3. Experimental Section

### 3.1. Reagents

Wafarin (W), acetyl-β-cyclodextrin (Ac-β-CD), methyl-β-cyclodextrin (Me-β-CD), and (2-hydroxyethyl)-β-cyclodextrin (HE-β-CD) (purity 99%) were purchased from Sigma-Aldrich Chemie GmbH (Taufkirchen, Germany). Millipore water used to prepare the solutions had a conductivity of less than 0.05 μS. The pH values of the solutions were adjusted (±0.2 units) by adding adequate amounts of HCl or NaOH.

### 3.2. Absorption/Steady-State Fluorescence Spectroscopy

The UV-visible absorption spectra were measured between 200 and 500 nm on a Cary-300 instrument (Agilent, Santa Clara, CA, USA) at room temperature. Fluorescence spectra measurements were scanned at room temperature, between 300 and 550 nm on a Cary-Eclipse fluorimeter. Slit widths were 5 nm for both excitation and emission monochromators, unless otherwise specified. The pH values were recorded using a pH meter (WTW 330i equipped with a WTW SenTix Mic (Xylem Analytics Germany Sales GmbH & Co., KG, WTW, Weilheim, Germany) Quartz cuvettes (1.0 cm, 4.0 mL) were used in all spectroscopic measurements and were obtained from Starna Cells Inc. (Atascadero, CA, USA). 

### 3.3. pH-Titration Studies

The pH titration by UV-visible absorption spectroscopic method was accomplished by measuring the pH values of about 3 mL solutions contained in a rectangular quartz cuvette with 1-cm optical path length, and the absorption spectra were then recorded. The concentrations of W and Me-β-CD were 25 μM and 3.5 mM, respectively. To adjust the pH, microliter volumes from 0.01 and 0.1 M NaOH and HCl solutions were pipetted consecutively to achieve the indicated pH values. The p*K*_a_ value was determined finally from fitting the titration data at a selected wavelength to a sigmoidal formula derived from Henderson-Hasselbalch and Beer-Lambert laws. The fitting algorithm was provided by the SigmaPlot software (version 6.1; Systat Software, Inc., San Jose, CA, USA). 

### 3.4. Steady-State Binding Titration Studies

In the titration experiments, the total concentrations of the W were kept constant and that of the host was gradually increased. The pH of a certain volume of H_2_O was first adjusted to either ~3 or ~9 in which a stock solution of W was prepared to give a final concentration of 25 μM. A calculated weight of β-CD derivatives was added to the same solution of W to prepare the stock solution of the complex (3.5 mM). The solutions with the final concentrations of β-CD were prepared by gradually adding increment volumes of the complex’s stock solution to 2.4 mL of the free W directly in the quartz cuvettes. The absorption or fluorescence spectra were measured for each solution. The signals at certain wavelength were plotted as a function of host’s total concentrations. The intermolecular interaction between β-CD and W may be quantified by the affinity constant referred to as the association equilibrium (*K*):

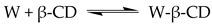
(1)

(2) $$K=\frac{\left[W-\beta -\mathrm{CD}\right]}{\left[W\right]\left[\beta -\mathrm{CD}\right]}$$

C_WF_ = [W] + [W-β-CD] (3)

C_β-CD_ = [β-CD] + [W-β-CD], (4)

where C_W_ and C_β-CD_ mean the total concentrations of W and β-CD, respectively. It can be written that:

Y (Reading at certain *λ*) = constant 1* [W] + constant 2* [W-β-CD] (5)

Using Equations (2)–(5), we obtain:

(6) $$\Delta Y=\frac{\Delta \left(\mathrm{constant}\right)C_{\beta -\mathrm{CD}}}{\frac{2}{KC_{W}-1-KC_{\beta -\mathrm{CD}}+\sqrt{{\left(1-KC_{W}+KC_{\beta -\mathrm{CD}}\right)}^{2}+4KC_{W}}}+1}$$

where ΔY = optical changes at a given λ; Δ(constant) = the difference between the molar absorptivity of free and β-CD-complexed W in the case of absorption titration, and *K* = binding constant. The binding constants (*K*) were then evaluated by using the nonlinear formula of Equation (6). Constant 2 was left as a floating parameter in the analysis by Levenberg-Marquardt algorithm, which was provided by the SigmaPlot software. 

### 3.5. Time-Resolved Fluorescence Measurements

The emission decays of W in the absence and presence of Me-β-CD at different pH values were collected using a LifeSpec II spectrometer (Edinburgh, Kirkton Campus, UK) based on the TCSPC method with excitation at 280 and 320 nm using two Edinburgh (Kirkton Campus, UK) laser diodes with repetition rate at 20 MHz and time resolution of ~90 picoseconds (ps). A red-sensitive high-speed PMT detector (H5773-04, Hamamatsu Photonics K. K., Hamamatsu, Japan) and a colour filter standard set (Edinburgh, Kirkton Campus, UK) with cut-off wavelength of 330 nm were used. Emission decays were collected every 10 nm over the entire emission spectra of W and W-β-CD complex in aqueous solution with a dwell time of 50 s at each wavelength. The concentration of W and Me-β-CD were 25 μM and 1.0 mM, respectively. The data were globally fitted with mono-exponential and bi-exponential model functions depending on the tested sample, then convoluted with instrument response function (IRF) of ~90 ps. The time-resolved data were specifically analyzed using Edinburgh FAST software (Figures S5–S10, Supporting Information) in which decay-associated spectra (DAS) were constructed from the extracted intensity-contribution fraction (*f*_i_) calculated from the pre-exponential amplitudes (*B_i_*), as follows:

(7) $$I\left(t\right)=\underset{i}{\sum }B_{i}exp\left(-t/\tau_{i}\right)$$

(8) $$f_{i}=\frac{B_{i}\tau_{i}}{\sum_{j}B_{j}\tau_{j}}$$

Target analysis utilizing the Glotaran software [19] were performed to confirm the kinetic expression for the population transfer of the two excited states that belong to free and CD-complexed W. Similar results were obtained by target analysis to those obtained by FAST (data not shown). The final results revealed that the two excited states decay monoexponentially in parallel, which validates the interpretation of DAS in our work as described above.

## 4. Conclusions

In this work, quantitative time-resolved fluorescence spectra of W measured using a picosecond diode laser with selective excitation of protonated isomers at 280 and 320 nm were conducted utilizing advanced lifetime analysis to give more specific information about the structures and kinetic behavior of excited-states of protonated and deprotonated drugs in water and inside CDs. We observed an increase in excited-state lifetime of the open protonated form upon its selective binding to Me-β-CD, over the cyclic form. The lifetime of the deprotonated form also increased upon inclusion. The increase in lifetime of the open protonated form was explained by radiative rate law, while that of the deprotonated form by energy-gap law. We have also demonstrated the higher acidity of the open form. With the aid of absorption and time-resolved fluorescence spectroscopies, we postulate the selective formation of hydrogen-bonded complexes in both the ground and excited states between the open form and the host. 

Fluorescence properties of W inside CDs in aqueous solutions at different pH values have attracted attention on several occasions due to their implications for bioanalytical quantification of W in commercial pesticides [12,20,21] and biological liquids [22]. Recent studies have also exploited W-CD systems as fluorescent probes and site markers in drug-protein interactions [23,24]. In addition to the fluorescence properties, better understanding of the interactions of W with CDs in the ground and excited states at different pH values should lead, in the future, to better modulation of drugs’ p*K*_a_ values and open-cyclic switchable properties in solution [10,11] and in different microheterogeneous environments that could advance their analytical separation by electrophoresis [15] and liquid chromatography [25,26], as well as their formulations [27] and clinical/biomedical applications [28]. Our results should, therefore, lead to better understanding of the role of CD on manipulating the open-cyclic switchable structure/function of this anticoagulant drug in ground and excited states. 

## Figures and Tables

**Table 1:** Spectroscopic and photophysical data of W at 25 µM in the absence and presence of Me-β-CD (1.0 mM). The DAS maximum for each lifetime is given in bracket.

Drug Forms	*λ*_abs_/nm	*ϵ*/M^−1^·cm^–1^	*λ*_ex_/nm	*λ*_em_/nm	*τ*_1_/ns (*λ*_em_/nm)	*τ*_2_/ns (*λ*_em_/nm)
Free (pH 3)	305	10 680	320	358	<0.1	
	282	12 820	280	358	<0.1	
Free (pH 9)	309	13 532	320	390	<0.1	
Complex (pH 3)	306	10 102	320 ^a^	358	<0.1 (358)	2.0 ± 0.4 ^b^ (375)
Complex (pH 9)	309	13 580	320	390	<0.1 (390)	1.22 ± 0.02 ^b^ (390)

^a^ With excitation at 280 nm, no change in excited-state lifetime was observed. ^b^ Values are presented as mean ± standard deviation. The estimated experimental error was 20% and 1.6% for the complex lifetimes at pH 3 and 9, respectively.

**Table 2:** Fluorescence quantum yield Ф_F_, radiative rate constant *k_r_*, and non-radiative rate constant *k_nr_* of different W species.

Drug Forms	*λ*_ex_/nm	Ф_F_ (×10^–3^) ^a^	*k_r_* (× 10^7^ s^–1^) ^b^	*k_nr_* (×10^9^ s^–1^) ^b^	*τ*/ns (Mean)
W OPEN	320	6.0 ± 0.1	>6	>10	<0.1
W^−^	320	12 ± 0.1	>12	>10	<0.1
W-Me-β-CD OPEN	320	0.5 ± 0.1	0.02	0.50	2.0
W^−^-Me-β-CD	320	24 ± 0.1	2.0	0.81	1.22

^a^ Measured using W in phosphate-buffered saline (PBS, pH ~7.4, 10 mM sodium phosphate) as the standard (Ф_F_ = 0.012) [9], and calculated using the known equation [18]: $\emptyset_{unk}=\emptyset_{std}\left(\frac{I_{unk}}{A_{unk}}\right)\left(\frac{A_{std}}{I_{std}}\right){\left(\frac{n_{unk}}{n_{std}}\right)}^{2}$, where *n* is the refractive indices for the standard (std) and experimental (unk) solvents, *I* is the fluorescence integral of the emission between 300 and 550 nm, and *A* is the absorbance at the excitation wavelength. The error estimated as standard deviation of the mean was approximately 10% for the fluorescence quantum yields. ^b^ Calculated using the known equations: $\emptyset =\frac{k_{r}}{k_{r}+k_{\mathrm{nr}}}$, $\tau =\frac{1}{k_{r}+k_{\mathrm{nr}}}$, $k_{\mathrm{nr}}=\frac{1-\emptyset }{\tau }$, and $k_{r}=\frac{\emptyset }{\tau }$.

## Supplementary Materials

The following are available online at www.mdpi.com/1420-3049/22/8/1326/s1, Figures S1–S10: Supporting Information.
