# Supplementary material for: Sequestration Effect on the Open-Cyclic Switchable Property of Warfarin Induced by Cyclodextrin: Time-Resolved Fluorescence Study
Source: Molecules. 2017 Aug 11;22(8):1326. doi: 10.3390/molecules22081326 (PMC6152132; doi:10.3390/molecules22081326)
Supplement: Supplementary file 1 [file molecules-22-01326-s001.pdf]

SUPPORTING INFORMATION

Sequestration Effect on the Open-Cyclic Switchable Property of Warfarin by Cyclodextrin: Time-Resolved Fluorescence Study

Naji Al-Dubaili and Na'il Saleh*

Chemistry Department, College of Science, United Arab Emirates University, P.O.Box 15551, Al-Ain, United Arab Emirates

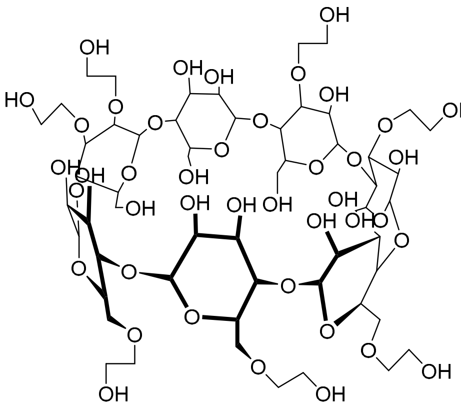

HE–β-CD

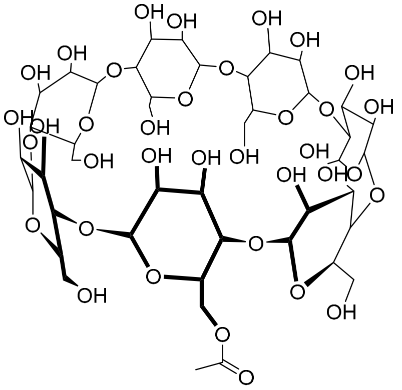

Ac–β-CD

Chart S1. The structures of selected *β*-cyclodextrins macrocycles in the present work that were not previously studied for their interactions with W.

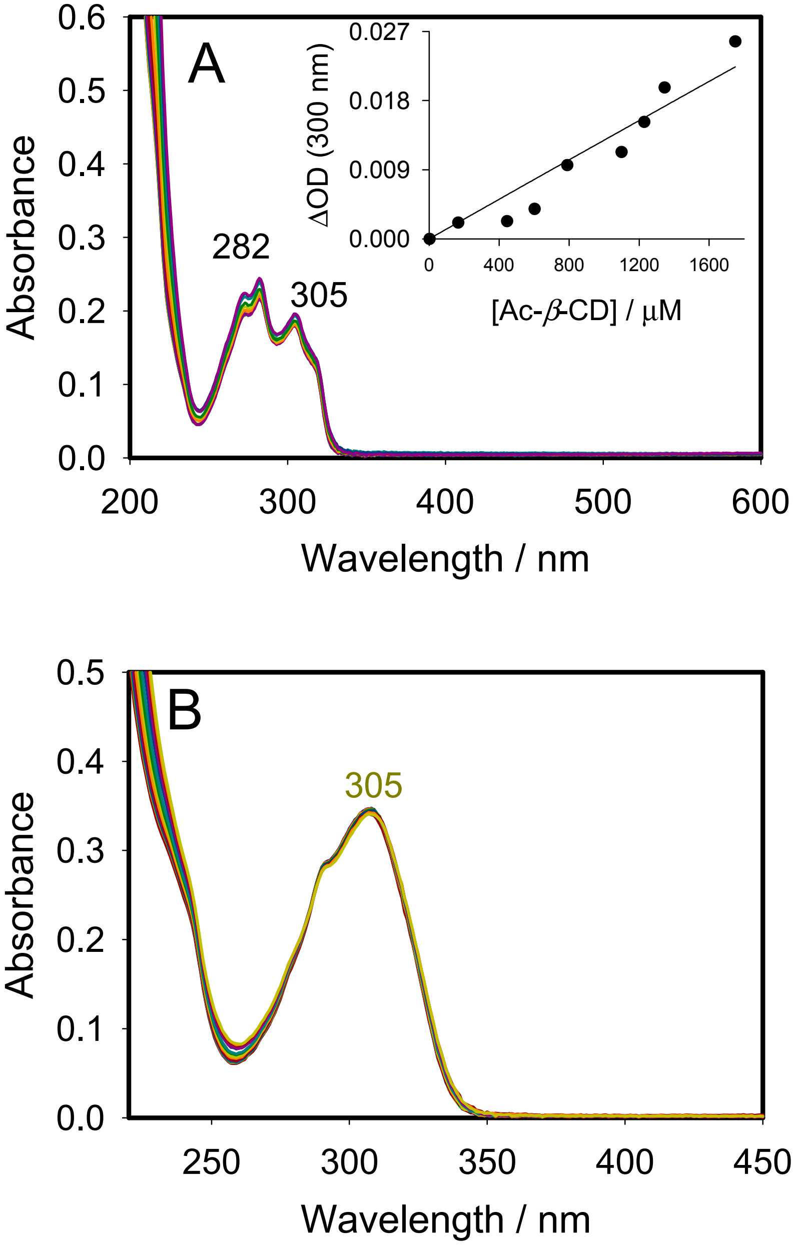

Figure S1. UV–Visible absorption titration of W (25 μM) with Ac-*β*-CD at pH 3 (A), and pH 9 (B); the *inset* shows the corresponding titration curve and the 1:1 binding fit (solid line) with K = (5.5 ± 78) M^{-1}. The very large error in binding affinity reflects very weak binding.

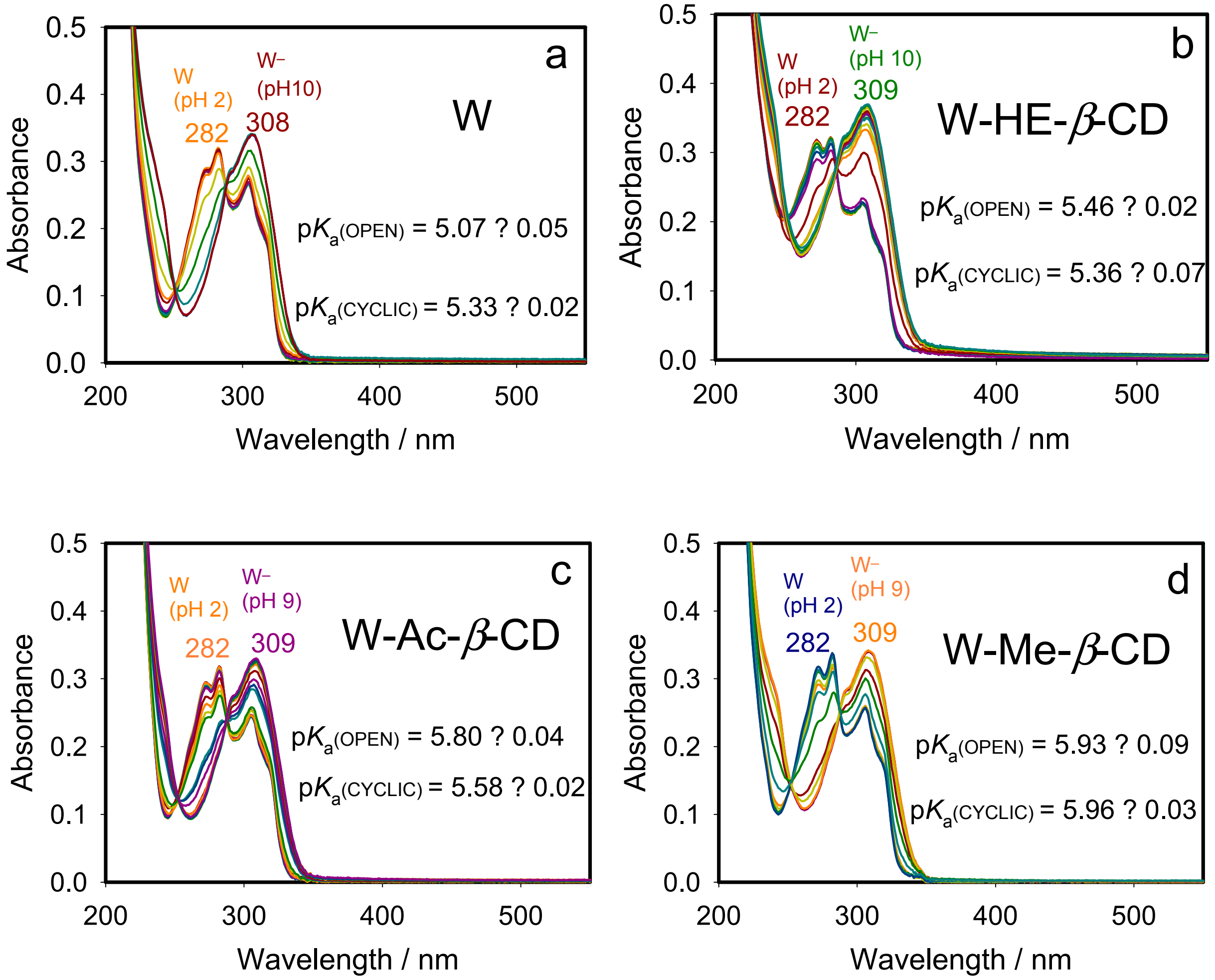

Figure S2. UV–visible absorption spectra at different pH values of W in water and inside HE-*β*-CD, Ac-*β*-CD, and Me-*β*-CD hosts. The sigmoidal fitting errors for each extracted pK_a at 280 (CYCLIC) and 320 nm (OPEN) are shown in the *insets*.

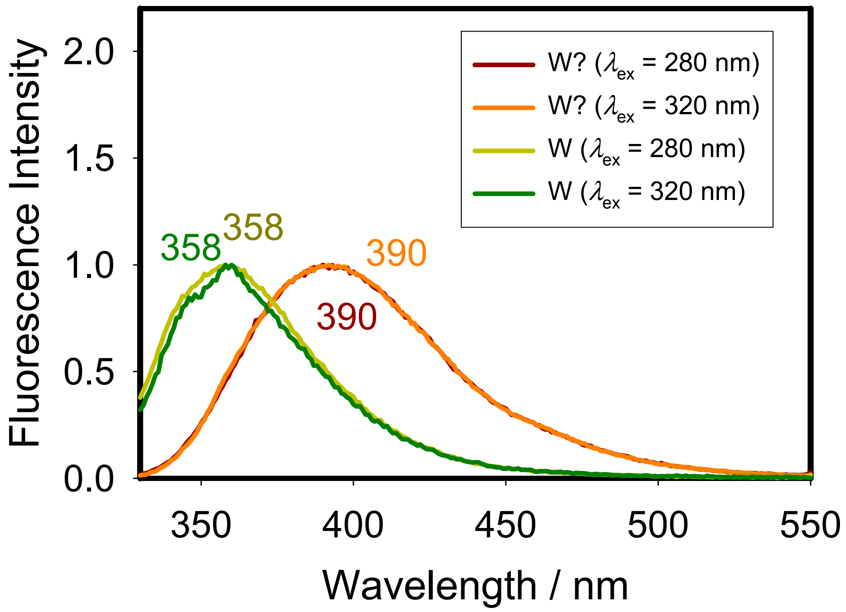

Figure S3. Fluorescence spectra of W (25 μM) at pH 3 and 9 with λ_{ex} = 320 nm and λ_{ex} = 280 nm as labeled by a distinct color. While no change in peak position, spectra of neutral W excited at 280 nm (dark yellow) and 320 nm (dark green) are different.

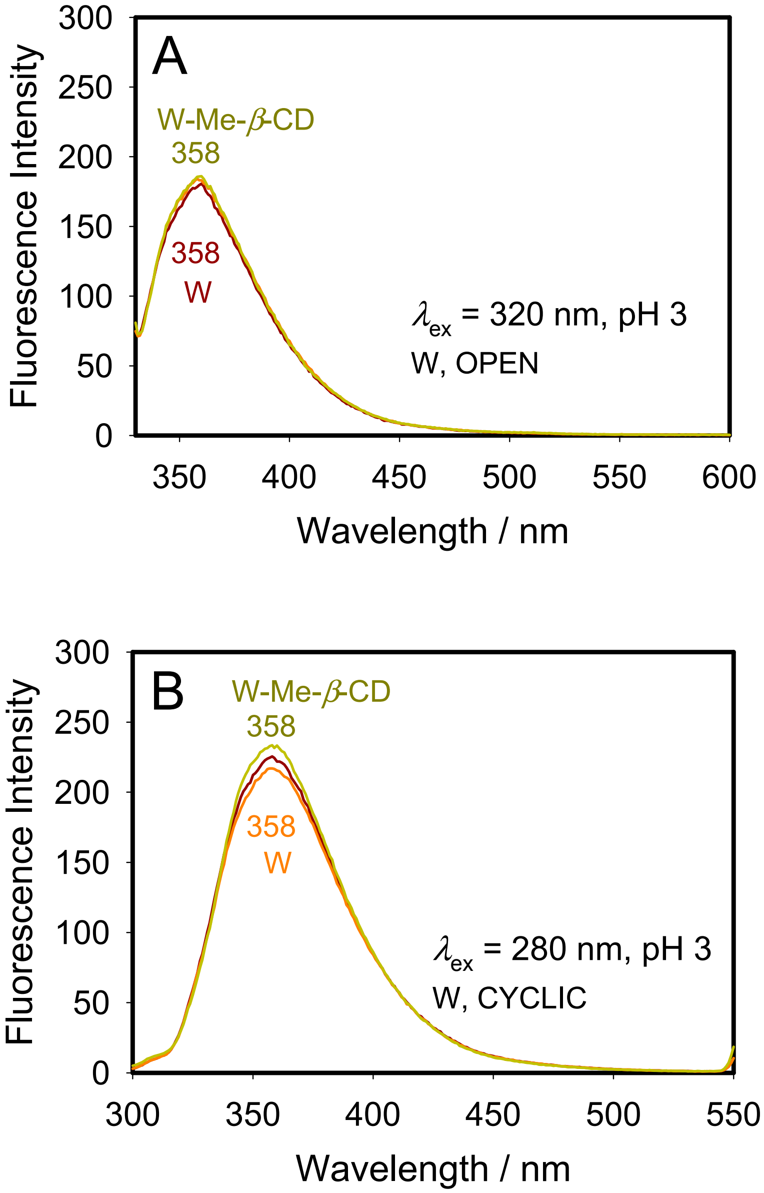

Figure S4. Fluorescence spectra of W (25 μM) at pH 3 upon the addition of Me-*β*-CD up to 250 μM (10 equiv.) with λ_{ex} = 320 nm (A); λ_{ex} = 280 nm (B), no significant changes in spectra were observed.

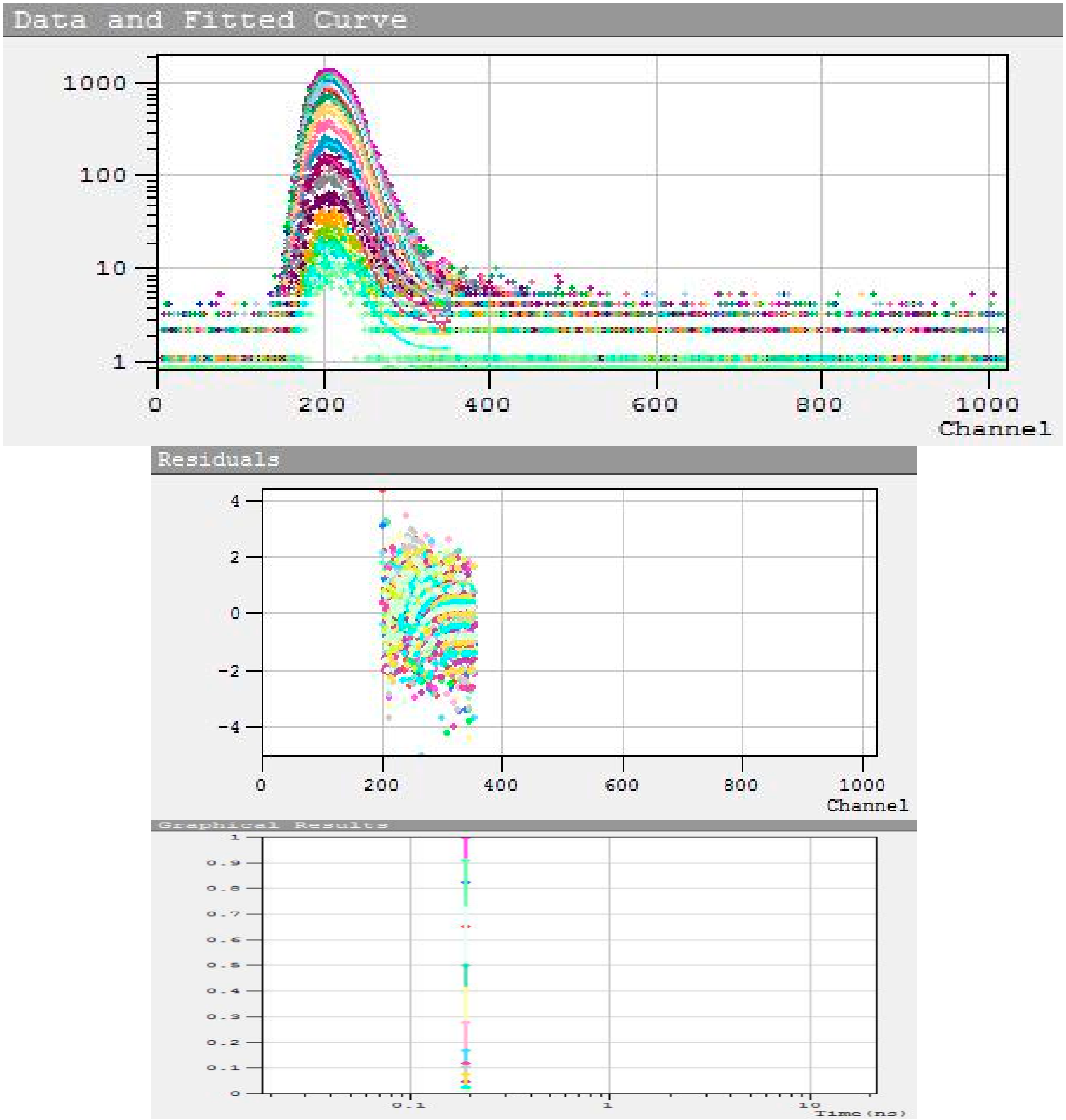

Figure S5. Collected emission decays measured over the emission spectrum of W at pH 3, excited at 320 nm from 330 to 490 nm every 10 nm with a dwell time of 50 s at each wavelength. Data at each wavelength were fitted to a mono-exponential model convoluted with IRF ~ 90 ps (as shown in the residuals), see Appendix I for fitting parameters.

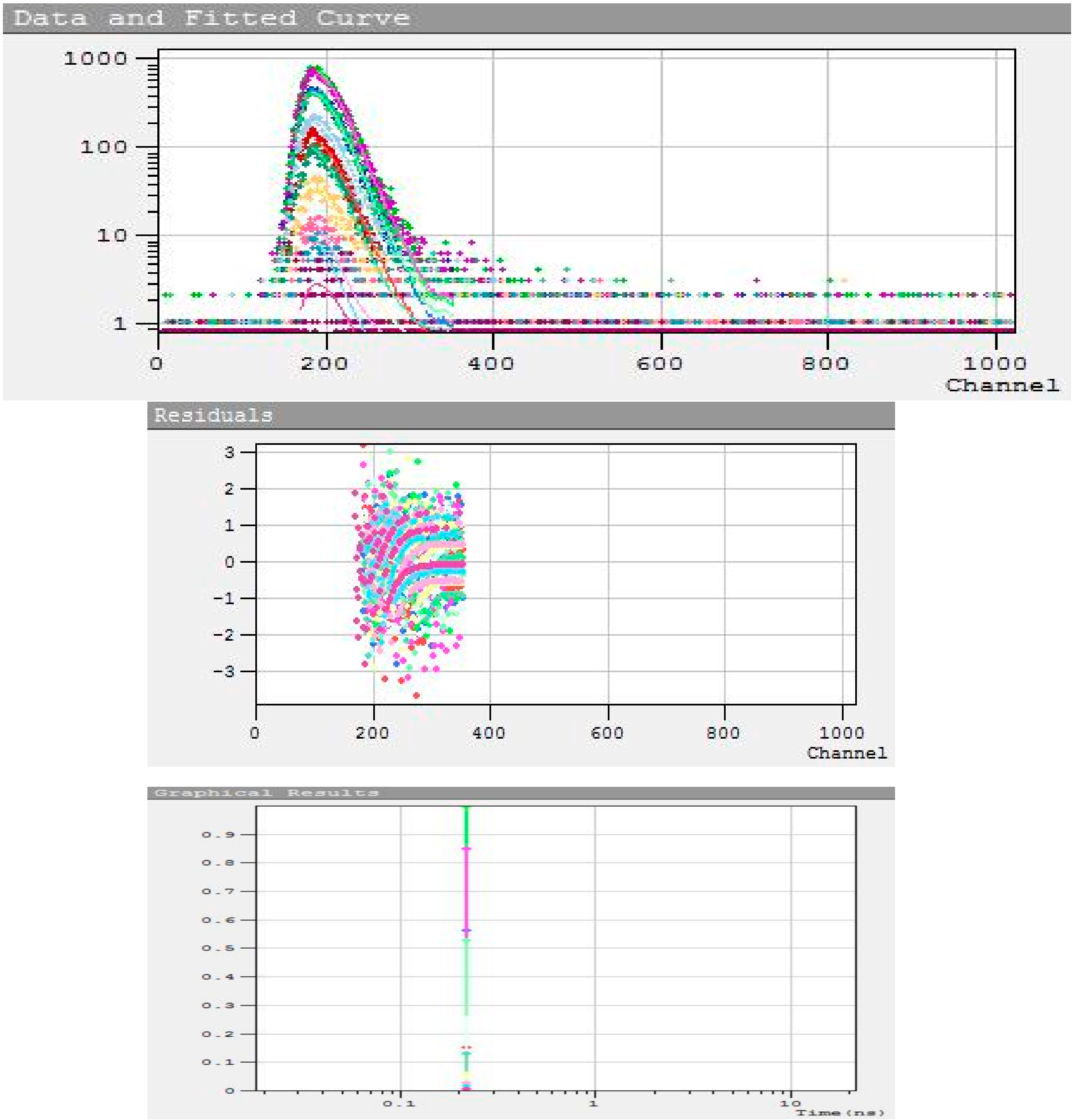

Figure S6. Collected emission decays measured over the emission spectrum of W at pH 3, excited at 280 nm from 310 to 510 nm every 20 nm with a dwell time of 50 s at each wavelength. Data at each wavelength were fitted to a mono-exponential model convoluted with IRF ~90 ps (as shown in the residuals, see Appendix II for fitting parameters.

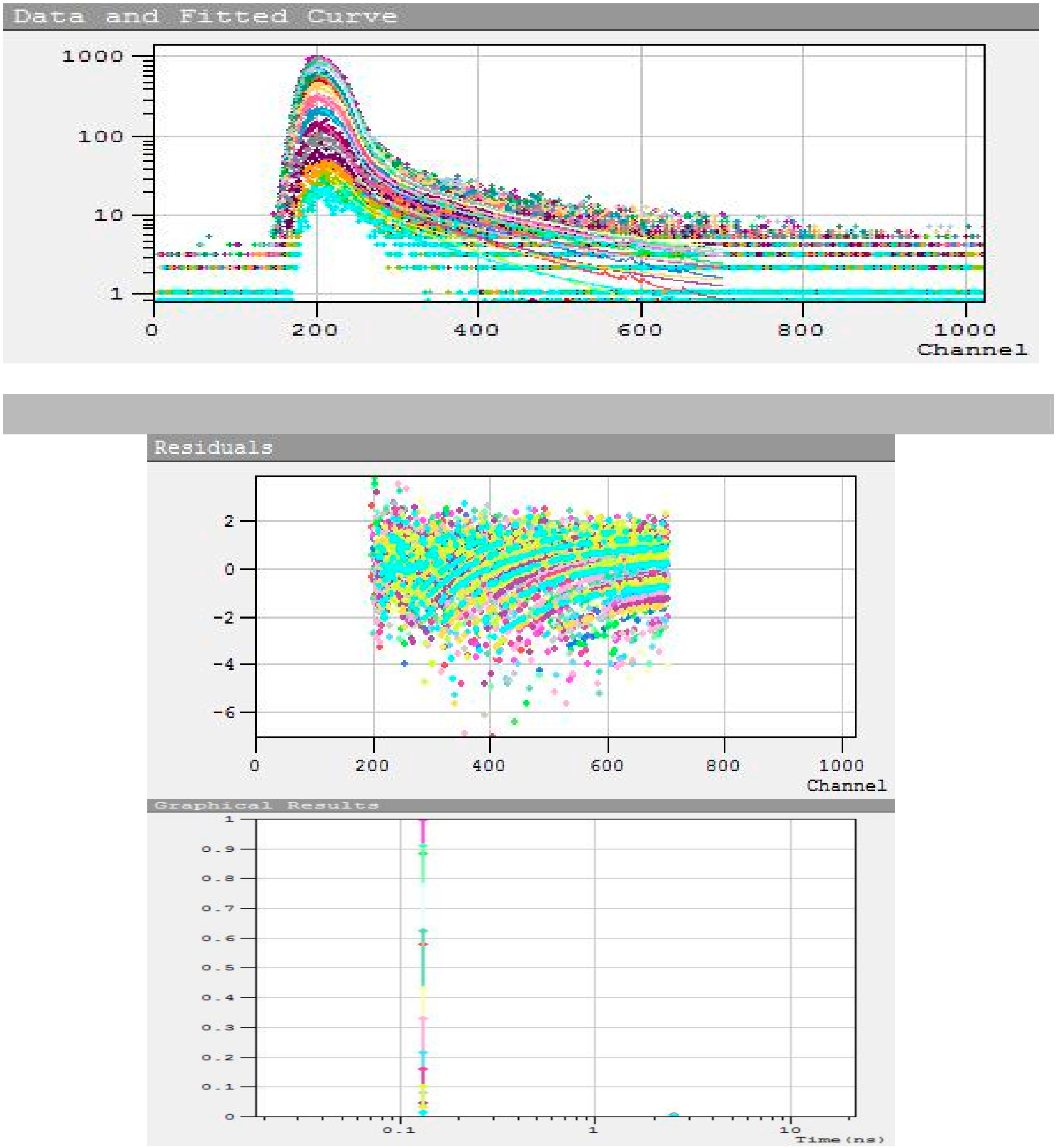

Figure S7. Collected emission decays measured over the emission spectrum of W-Me-β-CD at pH 3, excited at 320 nm (Figure 6A) from 330 to 480 nm every 10 nm with a dwell time of 50 s at each wavelength. Data at each wavelength were fitted to a bi-exponential model convoluted with IRF ~ 90 ps (as shown in the residuals), assuming 2 excited states that decay mono-exponentially in parallel (Figure 8A),[11] see Appendix III for fitting parameters.

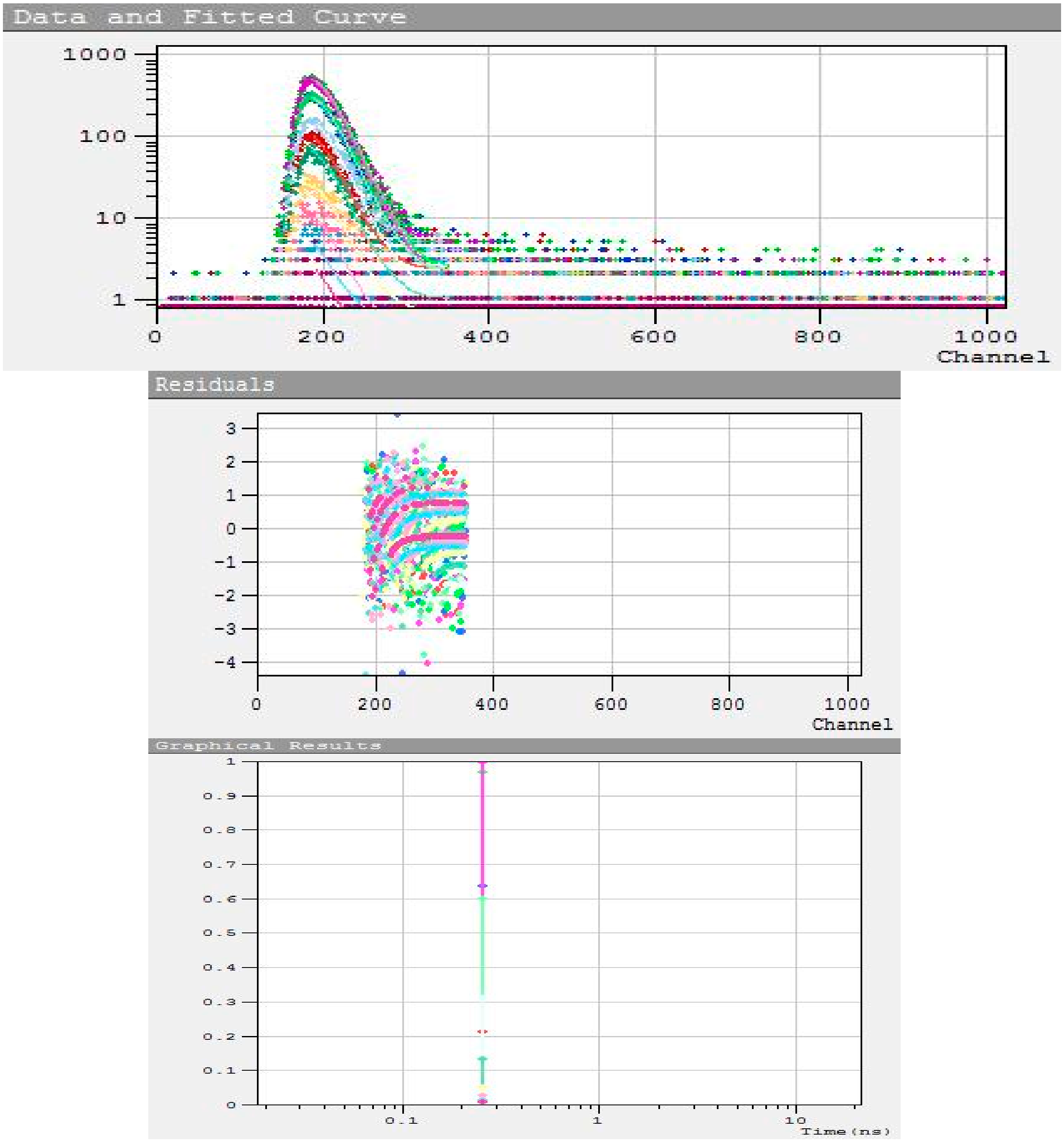

Figure S8. Collected emission decays measured over the emission spectrum of W-Me-*β*-CD at pH 3, excited at 280 nm from 310 to 510 nm every 20 nm with a dwell time of 50 s at each wavelength. Data at each wavelength were fitted to a mono-exponential model convoluted with IRF ~ 90 ps (as shown in the residuals), see Appendix IV.

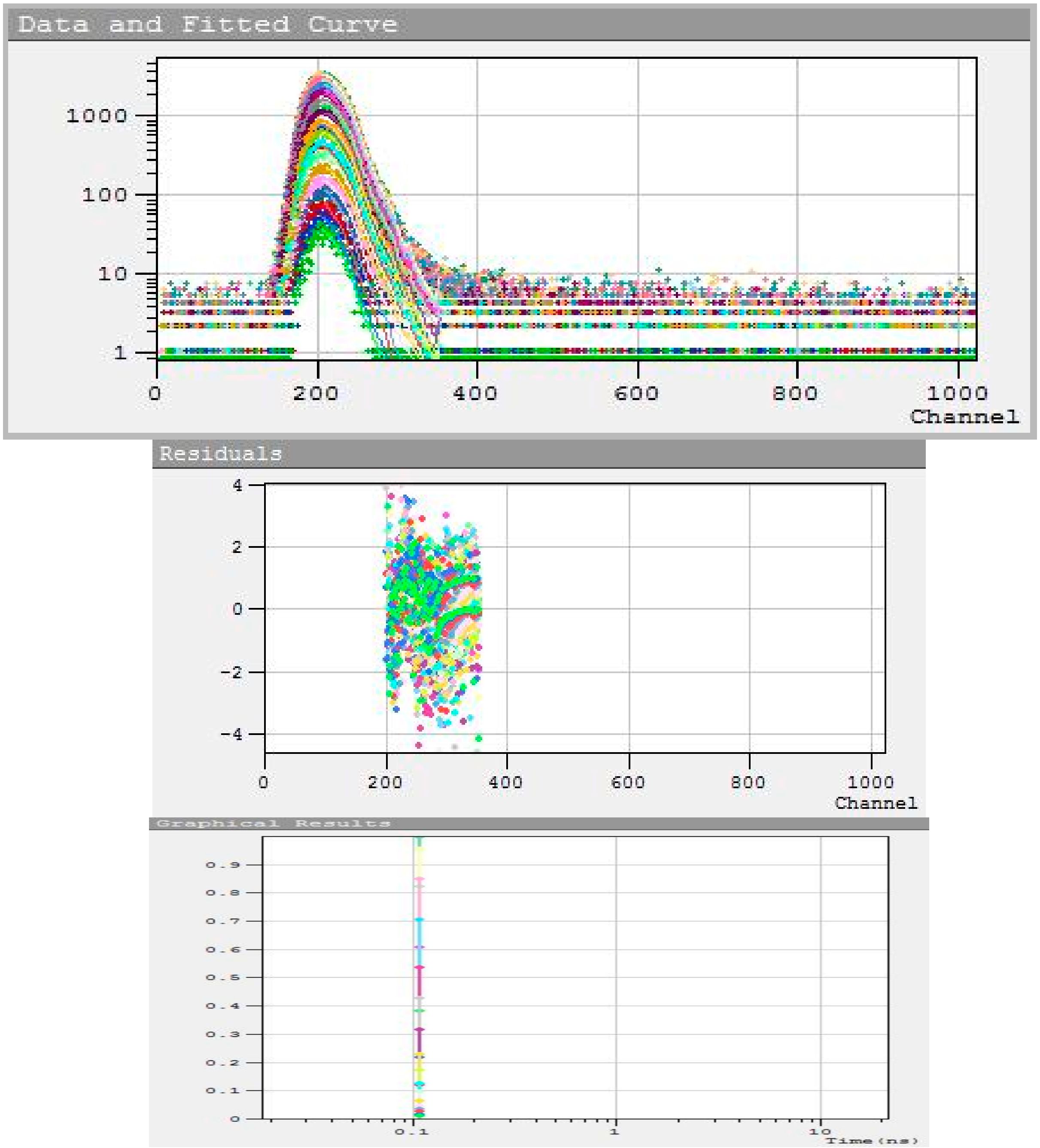

Figure S9. Collected emission decays measured over the emission spectrum of W at pH 9, excited at 320 nm from 330 to 550 nm every 10 nm with a dwell time of 50 s at each wavelength. Data at each wavelength were fitted to a mono-exponential model convoluted with IRF ~ 90 ps (as shown in the residuals), see Appendix V for fitting parameters.

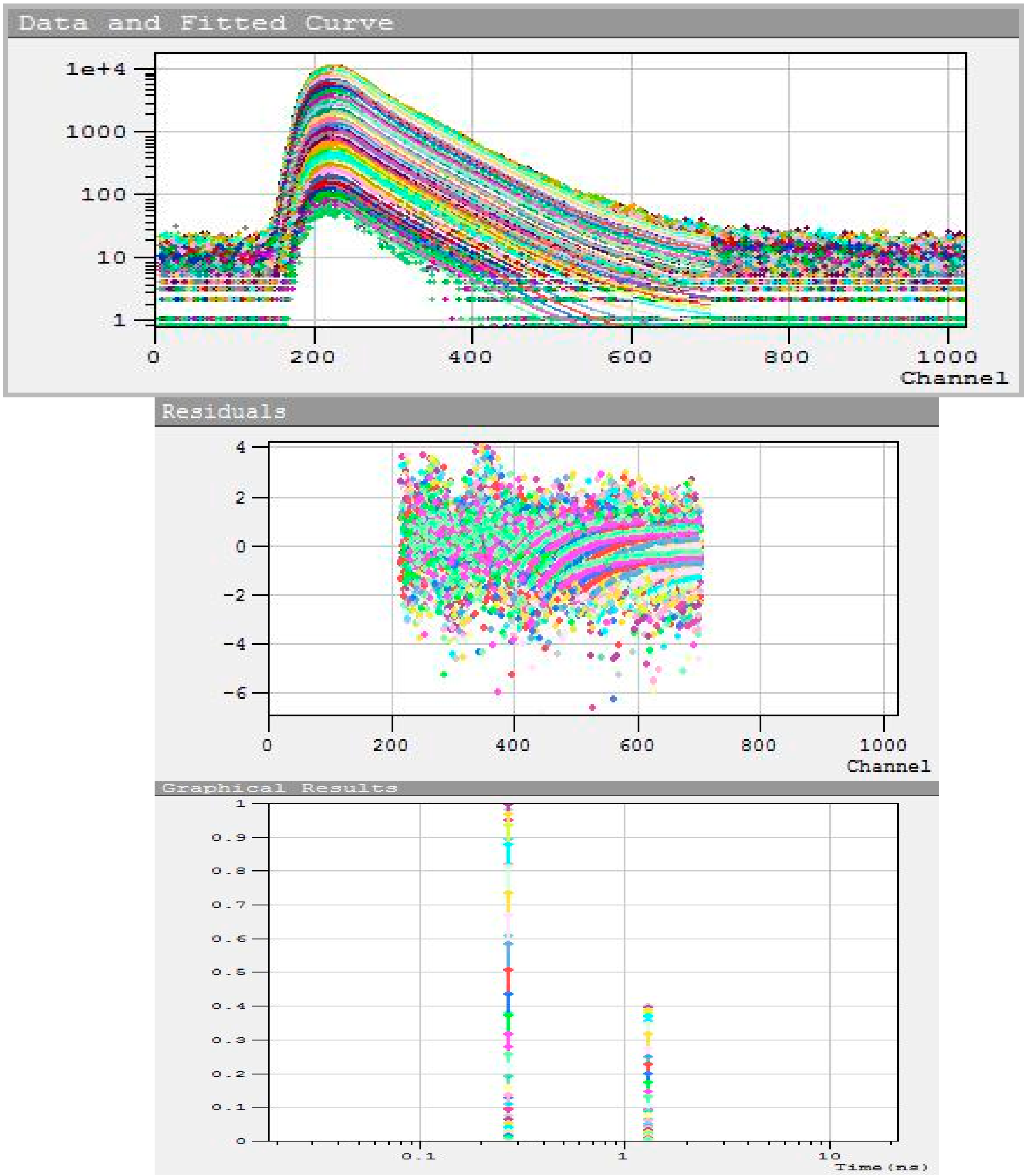

Figure S10. Collected emission decays measured over the emission spectrum of W-Me-β-CD at pH 9, excited at 320 nm (Figure 6B) from 330 to 550 nm every 5 nm with a dwell time of 50 s at each wavelength. Data at each wavelength were fitted to a bi-exponential model convoluted with IRF~ 90 ps (as shown in the residuals), assuming 2 excited states that decay bi-exponentially in parallel (Figure 8B),[11] see Appendix VI.

APPENDIX I

Global Analysis Data of W (pH 3; 320 nm)

File: Em1=330.00nm

❖ **Global Analysis (Reconvolution)**

Fitting range : [198; 350] channels

Global χ^2 : 1.330

χ^2 : 1.373

	B_i	f_i	τ_i (ns)
1	0.1193	100.00	0.182 linked

Shift : -0.0623 ns

Decay Background : 1.7676

IRF Background : 0.6000

File: Em1=340.00nm

❖ **Global Analysis (Reconvolution)**

Fitting range : [198; 350] channels

Global χ^2 : 1.330

χ^2 : 1.317

	B_i	f_i	τ_i (ns)
1	0.1511	100.00	0.182 linked

Shift : -0.0843 ns

Decay Background : 2.6230

IRF Background : 0.6000

File: Em1=350.00nm

❖ **Global Analysis (Reconvolution)**

Fitting range : [203; 350] channels
Global χ^2 : 1.330
χ^2 : 1.502

	B_i	f_i	τ_i (ns)
1	0.1835	100.00	0.182 linked

Shift : -0.0662 ns

Decay Background : 3.3868

IRF Background : 0.6000

File: Em1=360.00nm

❖ **Global Analysis (Reconvolution)**

Fitting range : [205; 350] channels
Global χ^2 : 1.330
χ^2 : 1.284

	B_i	f_i	τ_i (ns)
1	0.1830	100.00	0.182 linked

Shift : -0.0390 ns

Decay Background : 5.2076

IRF Background : 0.6000

File: Em1=370.00nm

❖ **Global Analysis (Reconvolution)**

Fitting range : [199; 350] channels
Global χ^2 : 1.330
χ^2 : 1.108

	B_i	f_i	τ_i (ns)
1	0.1667	100.00	0.182 linked

Shift : -0.0609 ns

Decay Background : 5.1108

IRF Background : 0.6000

File: Em1=380.00nm

❖ **Global Analysis (Reconvolution)**

Fitting range : [206; 350] channels

Global χ^2 : 1.330

χ^2 : 1.399

	B_i	f_i	τ_i (ns)
1	0.1327	100.00	0.182 linked

Shift : -0.0579 ns

Decay Background : 4.8014

IRF Background : 0.6000

File: Em1=390.00nm

❖ **Global Analysis (Reconvolution)**

Fitting range : [204; 350] channels

Global χ^2 : 1.330

χ^2 : 1.121

	B_i	f_i	τ_i (ns)
1	0.0915	100.00	0.182 linked

Shift : -0.0348 ns

Decay Background : 5.0537

IRF Background : 0.6000

File: Em1=400.00nm

❖ **Global Analysis (Reconvolution)**

Fitting range : [199; 350] channels
Global χ^2 : 1.330
χ^2 : 1.309

	B_i	f_i	τ_i (ns)
1	0.0742	100.00	0.182 linked

Shift : -0.0820 ns

Decay Background : 4.7219

IRF Background : 0.6000

File: Em1=410.00nm

- **Global Analysis (Reconvolution)**

Fitting range : [203; 350] channels
Global χ^2 : 1.330
χ^2 : 1.611

	B_i	f_i	τ_i (ns)
1	0.0505	100.00	0.182 linked

Shift : -0.0890 ns

Decay Background : 5.1625

IRF Background : 0.6000

File: Em1=420.00nm

- **Global Analysis (Reconvolution)**

Fitting range : [198; 350] channels
Global χ^2 : 1.330
χ^2 : 1.370

	B_i	f_i	τ_i (ns)
1	0.0305	100.00	0.182 linked

Shift : -0.0587 ns

Decay Background : 4.5170

IRF Background : 0.6000

File: Em1=430.00nm

❖ **Global Analysis (Reconvolution)**

Fitting range : [199; 350] channels
Global χ^2 : 1.330
χ^2 : 1.577

	B_i	f_i	τ_i (ns)
1	0.0214	100.00	0.182 linked

Shift : -0.1367 ns

Decay Background : 3.5061

IRF Background : 0.6000

File: Em1=440.00nm

❖ **Global Analysis (Reconvolution)**

Fitting range : [207; 350] channels
Global χ^2 : 1.330
χ^2 : 1.943

	B_i	f_i	τ_i (ns)
1	0.0187	100.00	0.182 linked

Shift : -0.2734 ns

Decay Background : 3.2706

IRF Background : 0.6000

File: Em1=450.00nm

❖ **Global Analysis (Reconvolution)**
Fitting range : [210; 350] channels

Global χ^2 : 1.330

χ^2 : 1.086

	B_i	f_i	τ_i (ns)
1	0.0079	100.00	0.182 linked

Shift : -0.0781 ns

Decay Background : 2.6210

IRF Background : 0.6000

File: Em1=460.00nm

❖ **Global Analysis (Reconvolution)**

Fitting range : [210; 350] channels

Global χ^2 : 1.330

χ^2 : 1.479

	B_i	f_i	τ_i (ns)
1	0.0136	100.00	0.182 linked

Shift : -0.5469 ns

Decay Background : 1.9734

IRF Background : 0.6000

File: Em1=470.00nm

❖ **Global Analysis (Reconvolution)**

Fitting range : [201; 350] channels

Global χ^2 : 1.330

χ^2 : 1.158

	B_i	f_i	τ_i (ns)
1	0.0032	100.00	0.182 linked

Shift : -0.0977 ns

Decay Background : 1.3457

IRF Background : 0.6000

File: Em1=480.00nm

Fitting range : [227; 350] channels

Global χ^2 : 1.330

χ^2 : 0.955

	B_i	f_i	τ_i (ns)
1	0.0041	100.00	0.182 linked

Shift : -0.2148 ns

Decay Background : 1.3984

IRF Background : 0.6000

File: Em1=490.00nm

❖ **Global Analysis (Reconvolution)**

Fitting range : [202; 350] channels

Global χ^2 : 1.330

χ^2 : 0.968

	B_i	f_i	τ_i (ns)
1	0.0018	100.00	0.182 linked

Shift : -0.2736 ns

Decay Background : 0.7885

IRF Background : 0.6000

APPENDIX II

Global Analysis Data of W (pH 3; 280 nm)

File: Em1=310.00nm

❖ **Global Analysis (Reconvolution)**

Fitting range : [182; 350] channels

Global χ^2 : 0.934

χ^2 : 1.185

	B_i	f_i	τ_i (ns)
1	0.0161	100.00	0.210 linked

Shift : -0.1562 ns

Decay Background : 0.6212

IRF Background : 0.3000

File: Em1=330.00nm

❖ **Global Analysis (Reconvolution)**

Fitting range : [178; 350] channels

Global χ^2 : 0.934

χ^2 : 0.935

	B_i	f_i	τ_i (ns)
1	0.0597	100.00	0.210 linked

Shift : -0.1969 ns

Decay Background : 0.7525

IRF Background : 0.3000

File: Em1=350.00nm

❖ **Global Analysis (Reconvolution)**

Fitting range : [186; 350] channels

Global χ^2 : 0.934

χ^2 : 1.057

	B_i	f_i	τ_i (ns)
1	0.1064	100.00	0.210 linked

Shift : -0.1953 ns

Decay Background : 1.3562

IRF Background : 0.3000

File: Em1=370.00nm

❖ **Global Analysis (Reconvolution)**

Fitting range : [182; 350] channels

Global χ^2 : 0.934

χ^2 : 1.427

	B_i	f_i	τ_i (ns)
1	0.0905	100.00	0.210 linked

Shift : -0.1325 ns

Decay Background : 1.6502

IRF Background : 0.3000

File: Em1=390.00nm

❖ **Global Analysis (Reconvolution)**

Fitting range : [180; 350] channels

Global χ^2 : 0.934

χ^2 : 0.949

	B_i	f_i	τ_i (ns)
1	0.0563	100.00	0.210 linked

Shift : -0.1777 ns

Decay Background : 1.0776

IRF Background : 0.3000

File: Em1=410.00nm

❖ **Global Analysis (Reconvolution)**

Fitting range : [184; 350] channels

Global χ^2 : 0.934

χ^2 : 1.151

	B_i	f_i	τ_i (ns)
1	0.0271	100.00	0.210 linked

Shift : -0.1758 ns

Decay Background : 1.1854

IRF Background : 0.3000

File: Em1=430.00nm

❖ **Global Analysis (Reconvolution)**

Fitting range : [180; 350] channels

Global χ^2 : 0.934

χ^2 : 0.963

	B_i	f_i	τ_i (ns)
1	0.0137	100.00	0.210 linked

Shift : -0.2871 ns

Decay Background : 0.8345

IRF Background : 0.3000

File: Em1=450.00nm

❖ Global Analysis (Reconvolution)

Fitting range : [188; 350] channels
Global χ^2 : 0.934
χ^2 : 0.842

	B_i	f_i	τ_i (ns)
1	0.0060	100.00	0.210 linked

Shift : -0.3125 ns

Decay Background : 0.5586

IRF Background : 0.3000

File: Em1=470.00nm

❖ Global Analysis (Reconvolution)

Fitting range : [187; 350] channels
Global χ^2 : 0.934
χ^2 : 0.765

	B_i	f_i	τ_i (ns)
1	0.0026	100.00	0.210 linked

Shift : -0.4883 ns

Decay Background : 0.5237

IRF Background : 0.3000

File: Em1=490.00nm

❖ Global Analysis (Reconvolution)

Fitting range : [185; 350] channels
Global χ^2 : 0.934
χ^2 : 0.503

	B_i	f_i	τ_i (ns)
1	0.0016	100.00	0.210 linked

Shift : -0.4687 ns

Decay Background : 0.2748

IRF Background : 0.3000

File: Em1=510.00nm

❖ Global Analysis (Reconvolution)

Fitting range : [166; 350] channels

Global χ^2 : 0.934

χ^2 : 0.524

	B_i	f_i	τ_i (ns)
1	0.0004	100.00	0.210 linked

Shift : -0.0504 ns

Decay Background : 0.0985

IRF Background : 0.3000

APPENDIX III

Global Analysis Data of W-Me-β-CD (pH 3; 320 nm)

File: Em1=330.00nm

❖ **Global Analysis (Reconvolution)**

Fitting range : [194; 700] channels
Global χ^2 : 1.302
χ^2 : 1.081

	B_i	f_i	τ_i (ns)
1	0.1037	92.15	0.127 linked
2	0.0005	7.8472	2.433 linked

Shift : -0.0757 ns

Decay Background : 0.4957

IRF Background : 0.6000

File: Em1=340.00nm

❖ **Global Analysis (Reconvolution)**

Fitting range : [198; 700] channels
Global χ^2 : 1.302
χ^2 : 1.130

	B_i	f_i	τ_i (ns)
1	0.1393	91.95	0.127 linked
2	0.0006	8.0530	2.433 linked

Shift : -0.0730 ns

Decay Background : 1.1128

IRF Background : 0.6000

File: Em1=350.00nm

❖ **Global Analysis (Reconvolution)**

Fitting range : [200; 700] channels
Global χ^2 : 1.302
χ^2 : 1.408

	B_i	f_i	τ_i (ns)
1	0.1586	91.31	0.127 linked
2	0.0008	8.6939	2.433 linked

Shift : -0.0112 ns

Decay Background : 1.8160

IRF Background : 0.6000

File: Em1=360.00nm

❖ **Global Analysis (Reconvolution)**

Fitting range : [198; 700] channels
Global χ^2 : 1.302
χ^2 : 1.418

	B_i	f_i	τ_i (ns)
1	0.1792	89.69	0.127 linked
2	0.0011	10.31	2.433 linked

Shift : -0.0514 ns

Decay Background : 2.0862

IRF Background : 0.6000

File: Em1=370.00nm

❖ **Global Analysis (Reconvolution)**

Fitting range : [200; 700] channels

Global χ^2 : 1.302

χ^2 : 1.274

	B_i	f_i	τ_i (ns)
1	0.1632	88.40	0.127 linked
2	0.0011	11.60	2.433 linked

Shift : -0.0391 ns

Decay Background : 2.7184

IRF Background : 0.6000

File: Em1=380.00nm

❖ Global Analysis (Reconvolution)

Fitting range : [201; 700] channels

Global χ^2 : 1.302

χ^2 : 1.341

	B_i	f_i	τ_i (ns)
1	0.1392	85.91	0.127 linked
2	0.0012	14.09	2.433 linked

Shift : -0.0510 ns

Decay Background : 2.2448

IRF Background : 0.6000

File: Em1=390.00nm

❖ Global Analysis (Reconvolution)

Fitting range : [196; 700] channels

Global χ^2 : 1.302

χ^2 : 1.419

	B_i	f_i	τ_i (ns)
1	0.1120	83.21	0.127 linked

2	0.0012	16.79	2.433 linked

Shift : -0.0977 ns

Decay Background : 2.3790

IRF Background : 0.6000

File: Em1=400.00nm

❖ Global Analysis (Reconvolution)

Fitting range : [202; 700] channels

Global χ^2 : 1.302

χ^2 : 1.278

	B_i	f_i	τ_i (ns)
1	0.0769	80.35	0.127 linked
2	0.0010	19.65	2.433 linked

Shift : -0.0427 ns

Decay Background : 3.1892

IRF Background : 0.6000

File: Em1=410.00nm

❖ Global Analysis (Reconvolution)

Fitting range : [200; 700] channels

Global χ^2 : 1.302

χ^2 : 1.624

	B_i	f_i	τ_i (ns)
1	0.0587	77.32	0.127 linked
2	0.0009	22.68	2.433 linked

Shift : -0.1172 ns

Decay Background : 2.6179

IRF Background : 0.6000

File: Em1=420.00nm

❖ **Global Analysis (Reconvolution)**

Fitting range : [203; 700] channels
Global χ^2 : 1.302
χ^2 : 1.381

	B_i	f_i	τ_i (ns)
1	0.0382	72.45	0.127 linked
2	0.0008	27.55	2.433 linked

Shift : -0.0781 ns

Decay Background : 2.4877

IRF Background : 0.6000

File: Em1=430.00nm

❖ **Global Analysis (Reconvolution)**

Fitting range : [208; 700] channels
Global χ^2 : 1.302
χ^2 : 1.387

	B_i	f_i	τ_i (ns)
1	0.0281	67.55	0.127 linked
2	0.0007	32.45	2.433 linked

Shift : -0.1953 ns

Decay Background : 1.6332

IRF Background : 0.6000

File: Em1=440.00nm

❖ **Global Analysis (Reconvolution)**

Fitting range : [204; 700] channels

Global χ^2 : 1.302
χ^2 : 1.370

	B_i	f_i	τ_i (ns)
1	0.0138	60.46	0.127 linked
2	0.0005	39.54	2.433 linked

Shift : -7e-6 ns

Decay Background : 1.9908

IRF Background : 0.6000

File: Em1=450.00nm

- **Global Analysis (Reconvolution)**

Fitting range : [204; 700] channels
Global χ^2 : 1.302
χ^2 : 1.142

	B_i	f_i	τ_i (ns)
1	0.0079	48.22	0.127 linked
2	0.0004	51.78	2.433 linked

Shift : 0.0459 ns

Decay Background : 0.8921

IRF Background : 0.6000

File: Em1=460.00nm

- **Global Analysis (Reconvolution)**

Fitting range : [207; 700] channels
Global χ^2 : 1.302
χ^2 : 1.258

	B_i	f_i	τ_i (ns)
1	0.0057	47.92	0.127 linked

2	0.0003	52.08	2.433 linked

Shift : -5e-6 ns

Decay Background : 1.2734

IRF Background : 0.6000

File: Em1=470.00nm

❖ Global Analysis (Reconvolution)

Fitting range : [219; 700] channels
Global χ^2 : 1.302
χ^2 : 1.246

	B_i	f_i	τ_i (ns)
1	0.0179	68.44	0.127 linked
2	0.0004	31.56	2.433 linked

Shift : -0.6250 ns

Decay Background : 0.1238

IRF Background : 0.6000

File: Em1=480.00nm

❖ Global Analysis (Reconvolution)

Fitting range : [206; 700] channels
Global χ^2 : 1.302
χ^2 : 1.062

	B_i	f_i	τ_i (ns)
1	0.0022	31.99	0.127 linked
2	0.0002	68.01	2.433 linked

Shift : -0.0195 ns

Decay Background : 0.5745

IRF Background : 0.6000

APPENDIX IV

Global Analysis Data of W-Me-β-CD (pH 3; 280 nm)

File: Em1=310.00nm

❖ **Global Analysis (Reconvolution)**

Fitting range : [184; 350] channels
Global χ^2 : 0.959
χ^2 : 1.118

	B_i	f_i	τ_i (ns)
1	0.0128	100.00	0.245 linked

Shift : -0.3125 ns

Decay Background : 2.4569

IRF Background : 0.3000

File: Em1=330.00nm

❖ **Global Analysis (Reconvolution)**

Fitting range : [184; 350] channels
Global χ^2 : 0.959
χ^2 : 1.222

	B_i	f_i	τ_i (ns)
1	0.0386	100.00	0.245 linked

Shift : -0.2734 ns

Decay Background : 2.8583

IRF Background : 0.3000

File: Em1=350.00nm

❖ **Global Analysis (Reconvolution)**

Fitting range : [184; 350] channels
Global χ^2 : 0.959
χ^2 : 1.121

	B_i	f_i	τ_i (ns)
1	0.0586	100.00	0.245 linked

Shift : -0.1808 ns

Decay Background : 2.4454

IRF Background : 0.3000

File: Em1=370.00nm

❖ **Global Analysis (Reconvolution)**

Fitting range : [182; 350] channels
Global χ^2 : 0.959
χ^2 : 1.300

	B_i	f_i	τ_i (ns)
1	0.0605	100.00	0.245 linked

Shift : -0.2498 ns

Decay Background : 2.0239

IRF Background : 0.3000

File: Em1=390.00nm

❖ **Global Analysis (Reconvolution)**

Fitting range : [183; 350] channels
Global χ^2 : 0.959
χ^2 : 1.262

	B_i	f_i	τ_i (ns)
1	0.0363	100.00	0.245 linked

Shift : -0.2144 ns

Decay Background : 2.2432

IRF Background : 0.3000

File: Em1=410.00nm

❖ **Global Analysis (Reconvolution)**

Fitting range : [189; 350] channels

Global χ^2 : 0.959

χ^2 : 0.951

	B_i	f_i	τ_i (ns)
1	0.0188	100.00	0.245 linked

Shift : -0.2539 ns

Decay Background : 1.4608

IRF Background : 0.3000

File: Em1=430.00nm

❖ **Global Analysis (Reconvolution)**

Fitting range : [183; 350] channels

Global χ^2 : 0.959

χ^2 : 0.968

	B_i	f_i	τ_i (ns)
1	0.0080	100.00	0.245 linked

Shift : -0.2532 ns

Decay Background : 1.0641

IRF Background : 0.3000

File: Em1=450.00nm

❖ **Global Analysis (Reconvolution)**

Fitting range : [175; 350] channels

Global χ^2 : 0.959

χ^2 : 0.689

	B_i	f_i	τ_i (ns)
1	0.0030	100.00	0.245 linked

Shift : -0.2539 ns

Decay Background : 0.7463

IRF Background : 0.3000

File: Em1=470.00nm

❖ **Global Analysis (Reconvolution)**

Fitting range : [191; 350] channels

Global χ^2 : 0.959

χ^2 : 0.850

	B_i	f_i	τ_i (ns)
1	0.0015	100.00	0.245 linked

Shift : -0.3907 ns

Decay Background : 0.4401

IRF Background : 0.3000

File: Em1=490.00nm

❖ **Global Analysis (Reconvolution)**

Fitting range : [180; 350] channels

Global χ^2 : 0.959

χ^2 : 0.673

	B_i	f_i	τ_i (ns)
1	0.0007	100.00	0.245 linked

Shift : -0.4688 ns

Decay Background : 0.5554

IRF Background : 0.3000

File: Em1=510.00nm

❖ **Global Analysis (Reconvolution)**

Fitting range : [187; 350] channels

Global χ^2 : 0.959

χ^2 : 0.398

	B_i	f_i	τ_i (ns)
1	0.0003	100.00	0.245 linked

Shift : -0.4297 ns

Decay Background : 0.2456

IRF Background : 0.3000

APPENDIX V

Global Analysis Data of W (pH 9; 320 nm)

File: Em1=330.00nm

❖ **Global Analysis (Reconvolution)**

Fitting range : [198; 350] channels

Global χ^2 : 1.436

χ^2 : 1.248

	B_i	f_i	τ_i (ns)
1	0.0992	100.00	0.104 linked

Shift : -0.0028 ns

Decay Background : 0.5518

IRF Background : 0.6000

File: Em1=340.00nm

❖ **Global Analysis (Reconvolution)**

Fitting range : [198; 350] channels

Global χ^2 : 1.436

χ^2 : 1.447

	B_i	f_i	τ_i (ns)
1	0.1835	100.00	0.104 linked

Shift : -0.0064 ns

Decay Background : 1.1423

IRF Background : 0.6000

File: Em1=350.00nm

❖ Global Analysis (Reconvolution)

Fitting range : [201; 350] channels
Global χ^2 : 1.436
χ^2 : 1.553

	B_i	f_i	τ_i (ns)
1	0.3226	100.00	0.104 linked

Shift : 0.0332 ns

Decay Background : 3.0877

IRF Background : 0.6000

File: Em1=360.00nm

❖ Global Analysis (Reconvolution)

Fitting range : [204; 350] channels
Global χ^2 : 1.436
χ^2 : 1.601

	B_i	f_i	τ_i (ns)
1	0.5130	100.00	0.104 linked

Shift : 0.0276 ns

Decay Background : 3.2082

IRF Background : 0.6000

File: Em1=370.00nm

❖ Global Analysis (Reconvolution)

Fitting range : [201; 350] channels
Global χ^2 : 1.436
χ^2 : 2.018

	B_i	f_i	τ_i (ns)

1	0.6966	100.00	0.104 linked

Shift : 0.0253 ns

Decay Background : 4.3292

IRF Background : 0.6000

File: Em1=380.00nm

❖ Global Analysis (Reconvolution)

Fitting range : [203; 350] channels
Global χ^2 : 1.436
χ^2 : 1.667

	B_i	f_i	τ_i (ns)
1	0.8048	100.00	0.104 linked

Shift : 0.0334 ns

Decay Background : 4.5986

IRF Background : 0.6000

File: Em1=390.00nm

❖ Global Analysis (Reconvolution)

Fitting range : [202; 350] channels
Global χ^2 : 1.436
χ^2 : 1.906

	B_i	f_i	τ_i (ns)
1	0.8458	100.00	0.104 linked

Shift : 0.0291 ns

Decay Background : 4.6974

IRF Background : 0.6000

File: Em1=400.00nm

❖ **Global Analysis (Reconvolution)**

Fitting range : [202; 350] channels
Global χ^2 : 1.436
χ^2 : 1.812

	B_i	f_i	τ_i (ns)
1	0.8080	100.00	0.104 linked

Shift : 0.0336 ns

Decay Background : 4.4687

IRF Background : 0.6000

File: Em1=410.00nm

❖ **Global Analysis (Reconvolution)**

Fitting range : [203; 350] channels
Global χ^2 : 1.436
χ^2 : 2.044

	B_i	f_i	τ_i (ns)
1	0.7190	100.00	0.104 linked

Shift : 0.0345 ns

Decay Background : 3.7058

IRF Background : 0.6000

File: Em1=420.00nm

❖ **Global Analysis (Reconvolution)**

Fitting range : [204; 350] channels
Global χ^2 : 1.436
χ^2 : 2.066

	B_i	f_i	τ_i (ns)

1	0.5974	100.00	0.104 linked

Shift : 0.0492 ns

Decay Background : 2.7790

IRF Background : 0.6000

File: Em1=430.00nm

❖ **Global Analysis (Reconvolution)**

Fitting range : [205; 350] channels
Global χ^2 : 1.436
χ^2 : 2.049

	B_i	f_i	τ_i (ns)
1	0.4522	100.00	0.104 linked

Shift : 0.0781 ns

Decay Background : 2.6920

IRF Background : 0.6000

File: Em1=440.00nm

❖ **Global Analysis (Reconvolution)**

Fitting range : [199; 350] channels
Global χ^2 : 1.436
χ^2 : 1.753

	B_i	f_i	τ_i (ns)
1	0.3605	100.00	0.104 linked

Shift : 0.0530 ns

Decay Background : 2.1747

IRF Background : 0.6000

File: Em1=450.00nm

❖ Global Analysis (Reconvolution)

Fitting range : [204; 350] channels
Global χ^2 : 1.436
χ^2 : 1.301

	B_i	f_i	τ_i (ns)
1	0.2664	100.00	0.104 linked

Shift : 0.0479 ns

Decay Background : 1.4656

IRF Background : 0.6000

File: Em1=460.00nm

❖ Global Analysis (Reconvolution)

Fitting range : [203; 350] channels
Global χ^2 : 1.436
χ^2 : 1.305

	B_i	f_i	τ_i (ns)
1	0.1934	100.00	0.104 linked

Shift : 0.0499 ns

Decay Background : 0.9652

IRF Background : 0.6000

File: Em1=470.00nm

❖ Global Analysis (Reconvolution)

Fitting range : [200; 350] channels
Global χ^2 : 1.436
χ^2 : 1.139

	B_i	f_i	τ_i (ns)

1	0.1461	100.00	0.104 linked

Shift : 0.0306 ns

Decay Background : 0.7773

IRF Background : 0.6000

File: Em1=480.00nm

❖ Global Analysis (Reconvolution)

Fitting range : [202; 350] channels

Global χ^2 : 1.436

χ^2 : 1.251

	B_i	f_i	τ_i (ns)
1	0.1048	100.00	0.104 linked

Shift : 0.0420 ns

Decay Background : 0.6611

IRF Background : 0.6000

File: Em1=490.00nm

❖ Global Analysis (Reconvolution)

Fitting range : [207; 350] channels

Global χ^2 : 1.436

χ^2 : 1.041

	B_i	f_i	τ_i (ns)
1	0.0796	100.00	0.104 linked

Shift : 0.0195 ns

Decay Background : 0.5155

IRF Background : 0.6000

File: Em1=500.00nm

❖ Global Analysis (Reconvolution)

Fitting range : [207; 350] channels
Global χ^2 : 1.436
χ^2 : 0.994

	B_i	f_i	τ_i (ns)
1	0.0534	100.00	0.104 linked

Shift : 0.0276 ns

Decay Background : 0.4548

IRF Background : 0.6000

File: Em1=510.00nm

❖ Global Analysis (Reconvolution)

Fitting range : [203; 350] channels
Global χ^2 : 1.436
χ^2 : 0.872

	B_i	f_i	τ_i (ns)
1	0.0366	100.00	0.104 linked

Shift : 0.0195 ns

Decay Background : 0.3143

IRF Background : 0.6000

File: Em1=520.00nm

❖ Global Analysis (Reconvolution)

Fitting range : [206; 350] channels
Global χ^2 : 1.436
χ^2 : 1.267

	B_i	f_i	τ_i (ns)
1	0.0290	100.00	0.104 linked

Shift : -0.0585 ns

Decay Background : 0.1086

IRF Background : 0.6000

File: Em1=530.00nm

❖ Global Analysis (Reconvolution)

Fitting range : [204; 350] channels
Global χ^2 : 1.436
χ^2 : 0.951

	B_i	f_i	τ_i (ns)
1	0.0204	100.00	0.104 linked

Shift : -0.1172 ns

Decay Background : 0.1034

IRF Background : 0.6000

File: Em1=540.00nm

❖ Global Analysis (Reconvolution)

Fitting range : [199; 350] channels
Global χ^2 : 1.436
χ^2 : 0.988

	B_i	f_i	τ_i (ns)
1	0.0132	100.00	0.104 linked

Shift : -0.1172 ns

Decay Background : 0.0268

IRF Background : 0.6000

File: Em1=550.00nm

- **Global Analysis (Reconvolution)**

Fitting range : [201; 350] channels

Global χ^2 : 1.436

χ^2 : 0.751

	B_i	f_i	τ_i (ns)
1	0.0089	100.00	0.104 linked

Shift : -0.0918 ns

Decay Background : 0.0312

IRF Background : 0.6000

APPENDIX VI

Global Analysis Data of W-Me-*β*-CD (pH 9; 320 nm)

File: Em1=330.00nm

❖ **Global Analysis (Reconvolution)**

Fitting range : [218; 700] channels

Global χ^2 : 1.253

χ^2 : 1.215

	B_i	f_i	τ_i (ns)
1	0.0524	41.84	0.259 linked
2	0.0151	58.16	1.248 linked

Shift : -0.0071 ns

Decay Background : 1.4812

IRF Background : 0.6000

File: Em1=335.00nm

❖ **Global Analysis (Reconvolution)**

Fitting range : [217; 700] channels

Global χ^2 : 1.253

χ^2 : 1.311

	B_i	f_i	τ_i (ns)
1	0.0705	39.78	0.259 linked
2	0.0222	60.22	1.248 linked

Shift : 0.0781 ns

Decay Background : 1.7349

IRF Background : 0.6000

File: Em1=340.00nm

❖ Global Analysis (Reconvolution)

Fitting range : [221; 700] channels

Global χ^2 : 1.253

χ^2 : 1.341

	B_i	f_i	τ_i (ns)
1	0.1063	38.57	0.259 linked
2	0.0352	61.43	1.248 linked

Shift : 0.0693 ns

Decay Background : 2.0643

IRF Background : 0.6000

File: Em1=345.00nm

❖ Global Analysis (Reconvolution)

Fitting range : [218; 700] channels

Global χ^2 : 1.253

χ^2 : 1.187

	B_i	f_i	τ_i (ns)
1	0.1558	38.86	0.259 linked
2	0.0510	61.14	1.248 linked

Shift : 0.1477 ns

Decay Background : 4.2210

IRF Background : 0.6000

File: Em1=350.00nm

❖ **Global Analysis (Reconvolution)**

Fitting range : [221; 700] channels

Global χ^2 : 1.253

χ^2 : 1.231

	B_i	f_i	τ_i (ns)
1	0.2122	37.53	0.259 linked
2	0.0734	62.47	1.248 linked

Shift : 0.1527 ns

Decay Background : 5.0772

IRF Background : 0.6000

File: Em1=355.00nm

❖ **Global Analysis (Reconvolution)**

Fitting range : [216; 700] channels

Global χ^2 : 1.253

χ^2 : 1.496

	B_i	f_i	τ_i (ns)
1	0.2801	36.23	0.259 linked
2	0.1025	63.77	1.248 linked

Shift : 0.1117 ns

Decay Background : 6.9354

IRF Background : 0.6000

File: Em1=360.00nm

❖ Global Analysis (Reconvolution)

Fitting range : [218; 700] channels
Global χ^2 : 1.253
χ^2 : 1.281

	B_i	f_i	τ_i (ns)
1	0.3405	35.73	0.259 linked
2	0.1273	64.27	1.248 linked

Shift : 0.1313 ns

Decay Background : 8.7025

IRF Background : 0.6000

File: Em1=365.00nm

❖ Global Analysis (Reconvolution)

Fitting range : [218; 700] channels
Global χ^2 : 1.253
χ^2 : 1.466

	B_i	f_i	τ_i (ns)
1	0.4045	35.49	0.259 linked
2	0.1528	64.51	1.248 linked

Shift : 0.1396 ns

Decay Background : 9.7300

IRF Background : 0.6000

File: Em1=370.00nm

❖ Global Analysis (Reconvolution)

Fitting range : [218; 700] channels

Global χ^2 : 1.253

χ^2 : 1.378

	B_i	f_i	τ_i (ns)
1	0.4601	35.08	0.259 linked
2	0.1770	64.92	1.248 linked

Shift : 0.1486 ns

Decay Background : 11.44

IRF Background : 0.6000

File: Em1=375.00nm

❖ **Global Analysis (Reconvolution)**

Fitting range : [217; 700] channels

Global χ^2 : 1.253

χ^2 : 1.585

	B_i	f_i	τ_i (ns)
1	0.5019	34.41	0.259 linked
2	0.1988	65.59	1.248 linked

Shift : 0.1444 ns

Decay Background : 12.98

IRF Background : 0.6000

File: Em1=380.00nm

❖ **Global Analysis (Reconvolution)**

Fitting range : [216; 700] channels

Global χ^2 : 1.253

χ^2 : 1.532

	B_i	f_i	τ_i (ns)
1	0.5336	33.83	0.259 linked
2	0.2168	66.17	1.248 linked

Shift : 0.1332 ns

Decay Background : 13.65

IRF Background : 0.6000

File: Em1=385.00nm

❖ Global Analysis (Reconvolution)

Fitting range : [221; 700] channels

Global χ^2 : 1.253

χ^2 : 1.455

	B_i	f_i	τ_i (ns)
1	0.5510	33.82	0.259 linked
2	0.2241	66.18	1.248 linked

Shift : 0.1514 ns

Decay Background : 14.16

IRF Background : 0.6000

File: Em1=390.00nm

❖ Global Analysis (Reconvolution)

Fitting range : [220; 700] channels

Global χ^2 : 1.253

χ^2 : 1.532

	B_i	f_i	τ_i (ns)
1	0.5608	34.54	0.259 linked

2	0.2209	65.46	1.248 linked

Shift : 0.1744 ns

Decay Background : 15.06

IRF Background : 0.6000

File: Em1=395.00nm

❖ **Global Analysis (Reconvolution)**

Fitting range : [222; 700] channels

Global χ^2 : 1.253

χ^2 : 1.612

	B_i	f_i	τ_i (ns)
1	0.5432	34.19	0.259 linked
2	0.2173	65.81	1.248 linked

Shift : 0.1929 ns

Decay Background : 15.69

IRF Background : 0.6000

File: Em1=400.00nm

❖ **Global Analysis (Reconvolution)**

Fitting range : [223; 700] channels

Global χ^2 : 1.253

χ^2 : 1.668

	B_i	f_i	τ_i (ns)
1	0.5264	34.02	0.259 linked
2	0.2121	65.98	1.248 linked

Shift : 0.1842 ns

Decay Background : 14.83

IRF Background : 0.6000

File: Em1=405.00nm

❖ Global Analysis (Reconvolution)

Fitting range : [221; 700] channels
Global χ^2 : 1.253
χ^2 : 1.434

	B_i	f_i	τ_i (ns)
1	0.4933	33.13	0.259 linked
2	0.2069	66.87	1.248 linked

Shift : 0.1658 ns

Decay Background : 14.43

IRF Background : 0.6000

File: Em1=410.00nm

❖ Global Analysis (Reconvolution)

Fitting range : [222; 700] channels
Global χ^2 : 1.253
χ^2 : 1.380

	B_i	f_i	τ_i (ns)
1	0.4553	32.71	0.259 linked
2	0.1947	67.29	1.248 linked

Shift : 0.1585 ns

Decay Background : 13.98

IRF Background : 0.6000

File: Em1=415.00nm

❖ Global Analysis (Reconvolution)

Fitting range : [220; 700] channels

Global χ^2 : 1.253

χ^2 : 1.511

	B_i	f_i	τ_i (ns)
1	0.4129	32.68	0.259 linked
2	0.1768	67.32	1.248 linked

Shift : 0.1779 ns

Decay Background : 12.56

IRF Background : 0.6000

File: Em1=420.00nm

❖ Global Analysis (Reconvolution)

Fitting range : [224; 700] channels

Global χ^2 : 1.253

χ^2 : 1.498

	B_i	f_i	τ_i (ns)
1	0.3752	33.82	0.259 linked
2	0.1526	66.18	1.248 linked

Shift : 0.2195 ns

Decay Background : 12.78

IRF Background : 0.6000

File: Em1=425.00nm

❖ Global Analysis (Reconvolution)

Fitting range : [226; 700] channels

Global χ^2 : 1.253

χ^2 : 1.397

	B_i	f_i	τ_i (ns)
1	0.3279	32.92	0.259 linked
2	0.1389	67.08	1.248 linked

Shift : 0.1953 ns

Decay Background : 11.28

IRF Background : 0.6000

File: Em1=430.00nm

❖ **Global Analysis (Reconvolution)**

Fitting range : [223; 700] channels

Global χ^2 : 1.253

χ^2 : 1.237

	B_i	f_i	τ_i (ns)
1	0.2836	31.93	0.259 linked
2	0.1257	68.07	1.248 linked

Shift : 0.1733 ns

Decay Background : 10.30

IRF Background : 0.6000

File: Em1=435.00nm

❖ **Global Analysis (Reconvolution)**

Fitting range : [217; 700] channels

Global χ^2 : 1.253

χ^2 : 1.181

	B_i	f_i	τ_i (ns)
1	0.2434	31.18	0.259 linked

2	0.1117	68.82	1.248 linked

Shift : 0.1482 ns

Decay Background : 8.7256

IRF Background : 0.6000

File: Em1=440.00nm

❖ Global Analysis (Reconvolution)

Fitting range : [220; 700] channels

Global χ^2 : 1.253

χ^2 : 1.037

	B_i	f_i	τ_i (ns)
1	0.2075	31.08	0.259 linked
2	0.0957	68.92	1.248 linked

Shift : 0.1525 ns

Decay Background : 8.1860

IRF Background : 0.6000

File: Em1=445.00nm

❖ Global Analysis (Reconvolution)

Fitting range : [221; 700] channels

Global χ^2 : 1.253

χ^2 : 1.289

	B_i	f_i	τ_i (ns)
1	0.1769	31.27	0.259 linked
2	0.0808	68.73	1.248 linked

Shift : 0.1634 ns

Decay Background : 7.4017

IRF Background : 0.6000

File: Em1=450.00nm

❖ **Global Analysis (Reconvolution)**

Fitting range : [216; 700] channels
Global χ^2 : 1.253
χ^2 : 1.363

	B_i	f_i	τ_i (ns)
1	0.1432	28.97	0.259 linked
2	0.0730	71.03	1.248 linked

Shift : 0.0997 ns

Decay Background : 6.2817

IRF Background : 0.6000

File: Em1=455.00nm

❖ **Global Analysis (Reconvolution)**

Fitting range : [219; 700] channels
Global χ^2 : 1.253
χ^2 : 1.175

	B_i	f_i	τ_i (ns)
1	0.1249	31.40	0.259 linked
2	0.0567	68.60	1.248 linked

Shift : 0.1771 ns

Decay Background : 4.9216

IRF Background : 0.6000

File: Em1=460.00nm

❖ **Global Analysis (Reconvolution)**

Fitting range : [217; 700] channels
Global χ^2 : 1.253
χ^2 : 1.175

	B_i	f_i	τ_i (ns)
1	0.1067	31.15	0.259 linked
2	0.0490	68.85	1.248 linked

Shift : 0.1482 ns

Decay Background : 4.3856

IRF Background : 0.6000

File: Em1=465.00nm

❖ **Global Analysis (Reconvolution)**

Fitting range : [225; 700] channels
Global χ^2 : 1.253
χ^2 : 1.142

	B_i	f_i	τ_i (ns)
1	0.0880	30.09	0.259 linked
2	0.0425	69.91	1.248 linked

Shift : 0.1172 ns

Decay Background : 3.2145

IRF Background : 0.6000

File: Em1=470.00nm

❖ **Global Analysis (Reconvolution)**

Fitting range : [224; 700] channels
Global χ^2 : 1.253
χ^2 : 1.442

	B_i	f_i	τ_i (ns)

1	0.0746	31.85	0.259 linked
2	0.0332	68.15	1.248 linked

Shift : 0.1897 ns

Decay Background : 3.4595

IRF Background : 0.6000

File: Em1=475.00nm

❖ **Global Analysis (Reconvolution)**

Fitting range : [229; 700] channels
Global χ^2 : 1.253
χ^2 : 1.072

	B_i	f_i	τ_i (ns)
1	0.0606	31.22	0.259 linked
2	0.0278	68.78	1.248 linked

Shift : 0.1952 ns

Decay Background : 3.2892

IRF Background : 0.6000

File: Em1=480.00nm

❖ **Global Analysis (Reconvolution)**

Fitting range : [226; 700] channels
Global χ^2 : 1.253
χ^2 : 1.242

	B_i	f_i	τ_i (ns)
1	0.0512	31.71	0.259 linked
2	0.0229	68.29	1.248 linked

Shift : 0.2066 ns

Decay Background : 2.3549

IRF Background : 0.6000

File: Em1=485.00nm

❖ Global Analysis (Reconvolution)

Fitting range : [223; 700] channels

Global χ^2 : 1.253

χ^2 : 1.200

	B_i	f_i	τ_i (ns)
1	0.0409	28.09	0.259 linked
2	0.0218	71.91	1.248 linked

Shift : 0.0804 ns

Decay Background : 2.3291

IRF Background : 0.6000

File: Em1=490.00nm

❖ Global Analysis (Reconvolution)

Fitting range : [212; 700] channels

Global χ^2 : 1.253

χ^2 : 1.314

	B_i	f_i	τ_i (ns)
1	0.0357	29.66	0.259 linked
2	0.0176	70.34	1.248 linked

Shift : 0.0956 ns

Decay Background : 2.0109

IRF Background : 0.6000

File: Em1=495.00nm

❖ Global Analysis (Reconvolution)

Fitting range : [227; 700] channels
Global χ^2 : 1.253
χ^2 : 1.130

	B_i	f_i	τ_i (ns)
1	0.0298	30.73	0.259 linked
2	0.0140	69.27	1.248 linked

Shift : 0.1367 ns

Decay Background : 1.7137

IRF Background : 0.6000

File: Em1=500.00nm

❖ Global Analysis (Reconvolution)

Fitting range : [218; 700] channels
Global χ^2 : 1.253
χ^2 : 1.068

	B_i	f_i	τ_i (ns)
1	0.0229	28.46	0.259 linked
2	0.0119	71.54	1.248 linked

Shift : 0.1115 ns

Decay Background : 1.3398

IRF Background : 0.6000

File: Em1=505.00nm

❖ Global Analysis (Reconvolution)

Fitting range : [215; 700] channels
Global χ^2 : 1.253
χ^2 : 1.106

	B_i	f_i	τ_i (ns)

1	0.0210	32.54	0.259 linked
2	0.0091	67.46	1.248 linked

Shift : 0.1656 ns

Decay Background : 1.0679

IRF Background : 0.6000

File: Em1=510.00nm

❖ **Global Analysis (Reconvolution)**

Fitting range : [221; 700] channels
Global χ^2 : 1.253
χ^2 : 1.121

	B_i	f_i	τ_i (ns)
1	0.0154	29.12	0.259 linked
2	0.0078	70.88	1.248 linked

Shift : 0.1367 ns

Decay Background : 1.3310

IRF Background : 0.6000

File: Em1=515.00nm

❖ **Global Analysis (Reconvolution)**

Fitting range : [228; 700] channels
Global χ^2 : 1.253
χ^2 : 1.079

	B_i	f_i	τ_i (ns)
1	0.0131	30.77	0.259 linked
2	0.0061	69.23	1.248 linked

Shift : 0.1454 ns

Decay Background : 0.7937

IRF Background : 0.6000

File: Em1=520.00nm

❖ Global Analysis (Reconvolution)

Fitting range : [221; 700] channels
Global χ^2 : 1.253
χ^2 : 1.008

	B_i	f_i	τ_i (ns)
1	0.0104	28.63	0.259 linked
2	0.0054	71.37	1.248 linked

Shift : 0.0298 ns

Decay Background : 0.8950

IRF Background : 0.6000

File: Em1=525.00nm

❖ Global Analysis (Reconvolution)

Fitting range : [215; 700] channels
Global χ^2 : 1.253
χ^2 : 0.979

	B_i	f_i	τ_i (ns)
1	0.0080	28.51	0.259 linked
2	0.0041	71.49	1.248 linked

Shift : 0.0675 ns

Decay Background : 0.6009

IRF Background : 0.6000

File: Em1=530.00nm

❖ Global Analysis (Reconvolution)

Fitting range : [211; 700] channels
Global χ^2 : 1.253
χ^2 : 1.048

	B_i	f_i	τ_i (ns)
1	0.0052	22.01	0.259 linked
2	0.0039	77.99	1.248 linked

Shift : -0.0679 ns

Decay Background : 0.3592

IRF Background : 0.6000

File: Em1=535.00nm

❖ **Global Analysis (Reconvolution)**

Fitting range : [224; 700] channels
Global χ^2 : 1.253
χ^2 : 0.924

	B_i	f_i	τ_i (ns)
1	0.0067	32.04	0.259 linked
2	0.0030	67.96	1.248 linked

Shift : -0.0719 ns

Decay Background : 0.3084

IRF Background : 0.6000

File: Em1=540.00nm

❖ **Global Analysis (Reconvolution)**

Fitting range : [216; 700] channels
Global χ^2 : 1.253
χ^2 : 0.803

	B_i	f_i	τ_i (ns)

1	0.0046	32.57	0.259 linked
2	0.0020	67.43	1.248 linked

Shift : 0.0888 ns

Decay Background : 0.2895

IRF Background : 0.6000

File: Em1=545.00nm

❖ Global Analysis (Reconvolution)

Fitting range : [227; 700] channels

Global χ^2 : 1.253

χ^2 : 0.878

	B_i	f_i	τ_i (ns)
1	0.0037	29.54	0.259 linked
2	0.0018	70.46	1.248 linked

Shift : -0.0247 ns

Decay Background : 0.4206

IRF Background : 0.6000

File: Em1=550.00nm

❖ Global Analysis (Reconvolution)

Fitting range : [228; 700] channels

Global χ^2 : 1.253

χ^2 : 0.847

	B_i	f_i	τ_i (ns)
1	0.0039	31.83	0.259 linked
2	0.0017	68.17	1.248 linked

Shift : -0.2150 ns

Decay Background : 0.2680

IRF Background : 0.6000
